# Mutations in the chloroplast inner envelope protein TIC100 impair and repair chloroplast protein import and impact retrograde signaling

**DOI:** 10.1093/plcell/koac153

**Published:** 2022-05-30

**Authors:** Naresh Loudya, Douglas P F Maffei, Jocelyn Bédard, Sabri Mohd Ali, Paul F Devlin, R Paul Jarvis, Enrique López-Juez

**Affiliations:** Department of Biological Sciences, Royal Holloway University of London, Egham TW20 0EX, UK; Department of Plant Sciences, University of Oxford, Oxford OX1 3RB, UK; Department of Biological Sciences, Royal Holloway University of London, Egham TW20 0EX, UK; Department of Plant Sciences, University of Oxford, Oxford OX1 3RB, UK; Department of Plant Sciences, University of Oxford, Oxford OX1 3RB, UK; Department of Biological Sciences, Royal Holloway University of London, Egham TW20 0EX, UK; Department of Plant Sciences, University of Oxford, Oxford OX1 3RB, UK; Department of Biological Sciences, Royal Holloway University of London, Egham TW20 0EX, UK

## Abstract

Chloroplast biogenesis requires synthesis of proteins in the nucleocytoplasm and the chloroplast itself. Nucleus-encoded chloroplast proteins are imported via multiprotein translocons in the organelle’s envelope membranes. Controversy exists around whether a 1-MDa complex comprising TIC20, TIC100, and other proteins constitutes the inner membrane TIC translocon. The *Arabidopsis thaliana cue8* virescent mutant is broadly defective in plastid development. We identify *CUE8* as *TIC100*. The *tic100^cue8^* mutant accumulates reduced levels of 1-MDa complex components and exhibits reduced import of two nucleus-encoded chloroplast proteins of different import profiles. A search for suppressors of *tic100^cue8^* identified a second mutation within the same gene, *tic100^soh1^*, which rescues the visible, 1 MDa complex-subunit abundance, and chloroplast protein import phenotypes. *tic100^soh1^* retains but rapidly exits virescence and rescues the synthetic lethality of *tic100^cue8^* when retrograde signaling is impaired by a mutation in the *GENOMES UNCOUPLED 1* gene. Alongside the strong virescence, changes in RNA editing and the presence of unimported precursor proteins show that a strong signaling response is triggered when TIC100 function is altered. Our results are consistent with a role for TIC100, and by extension the 1-MDa complex, in the chloroplast import of photosynthetic and nonphotosynthetic proteins, a process which initiates retrograde signaling.

## Teaser

Complementary mutations in TIC100 of the chloroplast inner envelope membrane cause reductions or corrective improvements in chloroplast protein import, and highlight a signaling role.

IN A NUTSHELL **Background:** Plants harvest energy from the sun and CO_2_ from the air and convert them into the energy-rich molecules they, and eventually us, are made of. Plants do this, photosynthesis, in bodies called chloroplasts inside their cells. Chloroplasts, made of protein and membrane material, were, before plants evolved, free-living bacteria, but the synthesis of most of their proteins occurs outside them, using information carried by the cell’s nuclear DNA, so most proteins have to be brought into developing chloroplasts, across the double membrane surrounding them, through dedicated, selective channels, formed by TOC (outer) and TIC (inner envelope) proteins. The identity of those channels matters as it helps determine versions of chloroplasts suited for particular environments. Which TIC proteins constitute the inner envelope channel has been a matter of controversy.

**Question:** A mutant Arabidopsis plant called *cue8* is slow-to-green (young leaves begin almost white) and shows delayed chloroplast and plant development. We looked for the molecular identity of the *CUE8* gene. We also caused further mutations in this mutant and searched whether any corrected the defects in *cue8*.

**Findings:** We found the mutated gene causing the *cue8* defects is the *TIC100* gene. This is one essential component of the “TIC 1-MDa complex,” one of the two versions of the TIC import complex under debate. That complex is made of several proteins, all present at reduced levels in *cue8*. In laboratory assays in which proteins are imported into isolated chloroplasts, *cue8* performed worse than normal plants for a photosynthetic and a housekeeping chloroplast protein. A corrective, “suppressor” mutant was identified, and it carried a second mutation in *TIC100*, one physically complementary to the first one. Both the single and the double (suppressed) mutant still were slow-to-green, which evidences a signaling role for import defects to the nucleus, making photosynthetic genes active or not.

**Next steps:** Surprisingly the grasses, including the cereals, have one core protein of the TIC 1 MDa complex but not the rest (including TIC100). We don’t know how their TIC channels operate. We also need to learn how the information on the defect in protein import, which occurs at the chloroplast envelope, is relayed to the cell’s nucleus (but we do have some clues).

## Introduction

Chloroplast-containing photosynthetic eukaryotes sustain the biosphere. Chloroplast biogenesis is a complex process which in plants requires the involvement of 2,000–3,000 nucleus-encoded proteins and ∼80 proteins encoded by the chloroplast’s own genome (Jarvis and López-Juez, 2013). The majority of proteins (those which are nucleus-encoded) need to be imported into the chloroplasts through the double-membrane envelope. This is achieved by the operation of protein import Translocons at the Outer and Inner envelope membrane of Chloroplasts, TOC and TIC respectively (Jarvis and López-Juez, 2013; Nakai, 2018; Richardson and Schnell, 2020).

At the outer membrane TOC complex, subunits with GTPase activity act as receptors for the N-terminal targeting signals of chloroplast-destined polypeptides, and another subunit, TOC75, acts as a transmembrane import channel (Jarvis and López-Juez, 2013). At least two versions of the TOC complex exist, with different client specificities: one contains receptors with a preference for abundant photosynthetic preproteins, while the other favors import of house-keeping preproteins, like those involved in the chloroplast genetic machinery (Ivanova et al., 2004; Kubis et al., 2004; Jarvis and López-Juez, 2013). Targeted replacement of TOC receptor proteins has been revealed as a fundamental determinant of the development of photosynthetic or nonphotosynthetic plastids and of plastid type transitions (Ling et al., 2012, 2019).

The identity of the inner membrane TIC components, in contrast, has been the subject of considerable debate. Initial studies identified an abundant 110 kDa protein (Kessler and Blobel, 1996; Lubeck et al., 1996), later named TIC110, and postulated to be a scaffold coordinating internal chaperones (Inaba et al., 2003) or, alternatively, the inner membrane import channel (Heins et al., 2002). Another candidate for the role of inner membrane channel is TIC20 (Chen et al., 2002; Kovacs-Bogdan et al., 2011), and a 1-MDa complex comprising nucleus-encoded TIC20 and at least three other proteins—including TIC100, TIC56, and chloroplast-encoded TIC214, but not including TIC110—has been identified as a core channel-forming TIC complex (Kikuchi et al., 2013). An alternative form of TIC20 was shown to occur in root tissue, in the absence of the other components of the complex. However, the 1-MDa TIC complex model has proven controversial (de Vries et al., 2015; Nakai, 2015b; Bolter and Soll, 2017; Sjuts et al., 2017; Richardson and Schnell, 2020). Objections center around the low abundance of TIC20 compared with TIC110 (Vojta et al., 2004; Kovacs-Bogdan et al., 2011), the fact that the three additional proteins of the 1-MDa complex are absent in the grass family (Kikuchi et al., 2013; de Vries et al., 2015), the observation that the full-length version of TIC56 is dispensable in *Arabidopsis thaliana* (Kohler et al., 2015, 2016; Schafer et al., 2019), and data that point to other functions for TIC56 and TIC214 (Kohler et al., 2016; Schafer et al., 2019). The observation of a combined TOC-TIC “supercomplex” which includes TIC20 but also a small fraction of the total TIC110 (Chen and Li, 2017), leaves the issue of the nature of the channel unresolved.

Chloroplast development in flowering plants occurs exclusively in the light (Arsovski et al., 2012), because photoreceptors activate the expression of many genes for chloroplast-destined proteins (Cackett et al., 2021). A genetic screen for mutants in which light failed to activate the promoter of the LHCB1*2 (*CAB3*) gene led to the identification of *CAB-underexpressed* (*cue*) mutants (Li et al., 1995; López-Juez et al., 1998). Among them, *cue8* exhibited a severe phenotype characterized by reduced plastid development in both dark and light conditions, and strongly impaired induction of (specifically) photosynthesis-associated genes by phytochrome photoreceptors (López-Juez et al., 1998; Vinti et al., 2005) linked to chloroplast-to-nucleus communication (Loudya et al., 2020). Seedlings of *cue8* display largely normal photomorphogenesis but have leaf rosettes with a virescent (slow-greening) phenotype. This virescence is due to a cellular correction phenomenon: an “anterograde” (nucleus-to-chloroplast) response which maintains a juvenile state of plastids, a delay in the transition from the pre-photosynthetic proplastid to differentiated chloroplast state, manifested in multiple physical and genetic features, and which allows for an eventual overcoming of the plastid defect (Loudya et al., 2020). This change is a response to “retrograde signals” (mediating chloroplast-to-nucleus communication) and can therefore be described as a “retro-anterograde correction.” Interestingly, evidence has recently accumulated pointing to an involvement of defects affecting the import of cytosol-synthesized proteins into chloroplasts (Wu et al., 2019; Tadini et al., 2020), or protein folding or quality control inside the organelle (Tadini et al., 2016; Wu et al., 2019; Tadini et al., 2020), in the initiation of changes in the cytosol. Such processes are broadly described as protein homeostasis or “proteostasis,” and their involvement triggers what can be described as a folding stress response, which may, in turn, cause retrograde signaling to the nucleus (Wu et al., 2019; Tadini et al., 2020). Indeed, GUN1, a chloroplast pentatricopeptide repeat protein which plays an important role in retrograde signaling, was shown to interact with chaperones involved in, or acting after, protein import, with its absence impairing import under specific conditions (Wu et al., 2019) or even depleting components of the import machinery itself (Tadini et al., 2020).

We sought the molecular identity of the *CUE8* gene by positional cloning. We here report that *cue8* carries a missense mutation affecting TIC100, one of the components of the 1-MDa TIC complex. Furthermore, a genetic screen for suppressors of this mutant identified a second, intragenic mutation. Comprehensive analyses of both *tic100^cue8^* and the suppressed mutant (carrying two mutations in the same gene) demonstrated a significant role for this protein in chloroplast protein import, which is in turn consistent with such a role for the 1-MDa complex. In spite of the suppressed mutant’s recovery in import capacity, it retained pronounced early virescence and exhibited strong genetic interaction with the loss of GUN1. Therefore, the results also highlighted the dramatic impact that changes in TIC100 have on chloroplast-to-nucleus communication.

## Results

### Mutation of *CUE8* leads to defects in plastid development in leaves and roots

**Figure 1:**
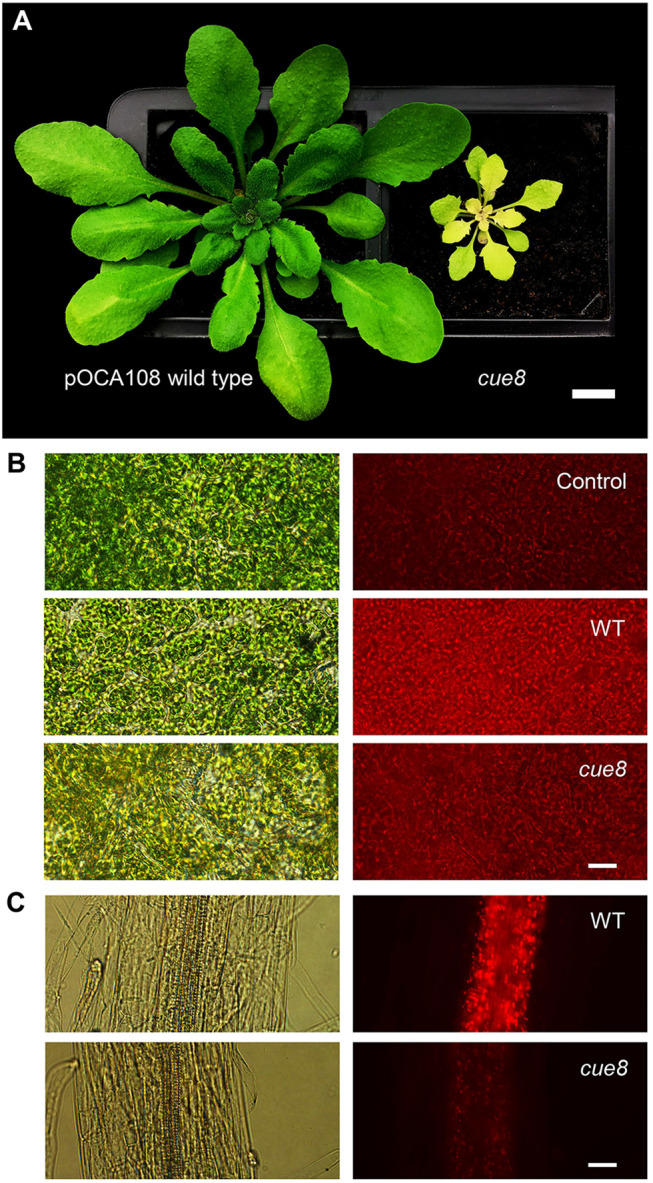
Mutation of *CUE8* causes a delay in plastid development in both aerial and root tissues. A, Phenotype of 28-day-old pOCA108 WT and *cue8* mutant plants. Scale bar 1 cm. B, Mature cotyledon samples of plants without plastid-targeted DsRed (Control), or DsRed-containing pOCA108 WT (5 days) or DsRed-containing *cue8* (6 days). C, Root samples of equivalent seedlings. The same transgene was present in both genotypes and images of WT and mutant were taken using the same exposure. Scale bar (B and C) 25 µm.

The *cue8* mutant was previously identified following mutagenesis of the pOCA108 reporter-containing line (Figure 1A; Li et al., 1995). We recently demonstrated that the virescent, slow-greening phenotype of *cue8* is associated with reduced chloroplast development in early cotyledons or very young leaf tissues (in which chloroplasts fail to fill the available cellular space), and by a gradual recovery of normal chloroplasts (Loudya et al., 2020). We wished to investigate whether the mutation influences plastid development beyond leaves, widely across tissues, and so incorporated a plastid-targeted DsRed fluorescent protein (Haswell and Meyerowitz, 2006) into the wild-type (WT), and then introgressed the transgene into the *cue8* mutant. The fluorescence signal was substantially reduced in *cue8*, relative to WT, both in cotyledon mesophyll cells and in roots, in which partially developed chloroplasts were prominent in cells surrounding the central vasculature (Figure 1, B and C). Accordingly, leaf development and root elongation were both reduced in the mutant (Supplemental Figure S1, A and B). Supplementation of the growth medium with sucrose rescued the *cue8* root phenotype, in a dose-dependent manner but to an incomplete extent (Supplemental Figure S1). Thus, we concluded that CUE8 plays a role in plastid development in nonphotosynthetic tissues, as well as in photosynthetic tissues.

### Identification of the *CUE8* locus by linkage mapping

**Figure 2:**
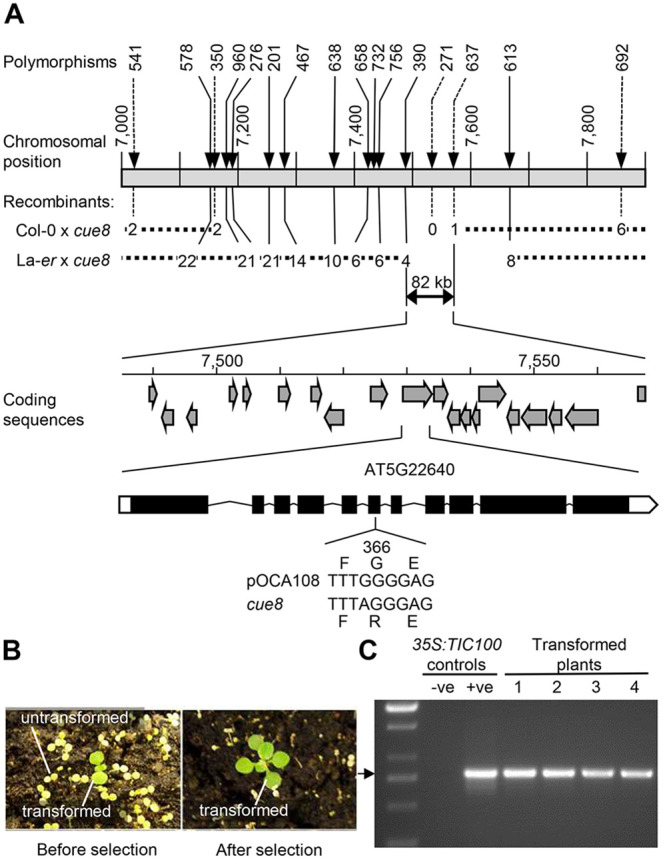
Cloning of *CUE8*. A, Map-based cloning of the *CUE8* gene, AT5G22640, encoding *TIC100*/*EMB1211*. Upper, abbreviated name of the polymorphisms used for mapping, position along chromosome 5 (in kb), and number of recombinants at those positions identified in the indicated mapping populations. This identifies an 82-kb region, containing 19 open-reading frames (middle). A combination of strategies (Supplemental Table S2) identifies AT5G22640 (*TIC100*) as the *CUE8* locus, whose exon/intron structure is shown. A point mutation (lower) results in a single amino acid substitution (G366R) in the TIC100 protein sequence. B, Complementation of *cue8* with *35S:TIC100*, carrying a *TIC100* cDNA under the control of a 35S promoter. Plants shown before and after the selection of transformants. C, Diagnostic PCR confirming the presence of the *35S:TIC100* transgene in complemented plants. Positive (+ve) control, plasmid DNA harboring the construct. Negative (−ve) control, DNA from plant prior to transformation.

*cue8* and its WT progenitor (Li et al., 1995) are lines in the Bensheim ecotype of Arabidopsis (Figure 1A). We generated two mapping populations for *cue8* by performing outcrosses to both Landsberg-*erecta* (La-*er*) and Columbia-0 (Col-0), to take advantage of ecotype polymorphisms (Supplemental Table S1). The *CUE8* locus was mapped to an 82-kb region of chromosome 5, containing 19 complete open reading frames (Figure 2A; see “Materials and methods”). A Transformation-competent Artificial Chromosome (TAC) covering 11 of those genes was able, when transformed into *cue8*, to complement the mutation (Supplemental Figure S2 and Supplemental Table S2). A combination of sequencing of individual candidate genes and assessment of the phenotypes of T-DNA knockouts (Supplemental Table S2) ruled out 10 of those genes, while we were unable to identify a viable homozygous mutant for AT5G22640 (only heterozygous T-DNA-containing plants were recovered). Sequencing of genomic DNA of *cue8* confirmed the presence of a mutation in this gene resulting in a G→R amino acid substitution at position 366, just outside one of the protein’s predicted Membrane Occupation and Recognition Nexus (MORN) domains (Takeshima et al., 2000; Figure 2A, Supplemental Table S3 and see below). Transformation of the mutant with a WT (pOCA108) cDNA encoded by AT5G22640 under the control of a constitutive promoter also resulted in complementation (Figure 2, B and C). Thus, we concluded that *CUE8* is AT5G22640, a gene identified previously as *EMB1211*, due to its embryo-lethal knockout mutant phenotype (Liang et al., 2010), and, most interestingly, as *TIC100* (Kikuchi et al., 2013), encoding a component of the putative 1-MDa TIC complex. We hereafter refer to the mutant allele, and the plant carrying it, as *tic100^cue8^*. Bearing in mind the nature of the *tic100^cue8^* amino acid substitution, as well as the mutant’s virescent phenotype, which contrasts with the loss of viability caused by a T-DNA insertion at this locus, we conclude that the *tic100^cue8^* is a hypomorphic allele, carrying a missense mutation which causes a partial loss-of-function of the gene.

Several chloroplast protein import components have previously been shown to have a preferential role in the import of either abundant, photosynthetic proteins or less-abundant, but essential, plastid housekeeping proteins (Jarvis, 2008). Having identified *CUE8*, we compared, using publicly available data (Schmid et al., 2005), its developmental expression with that of genes representative of those two functions. The *CUE8*/*TIC100* gene exhibited (Supplemental Figure S3) a combined expression pattern: high like *LHCB2.1* in photosynthetic tissues, while also high like *TOC34* in those tissues rich in meristematic cells, such as the root tip. Results of a search for co-regulated genes, using two different algorithms (Supplemental Data Sets S1 and S2) were also consistent with *CUE8*/*TIC100* being involved early (e.g. together with transcription and translation, pigment synthesis and protein import functions) in the biogenesis of photosynthetic as well as nonphotosynthetic plastids.

### Reduced protein import rate and partial loss of the 1-MDa complex in *tic100^cue8^* chloroplasts

**Figure 3:**
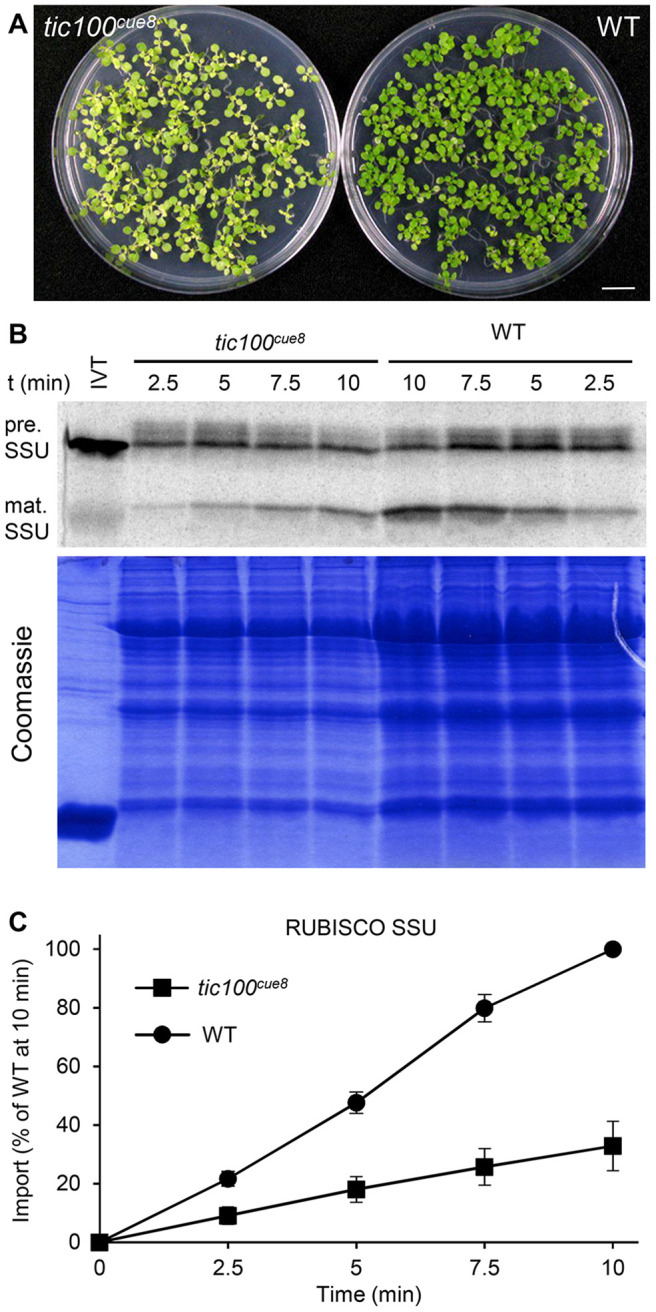
Chloroplasts of *tic100^cue^^8^* exhibit reduced protein import rates. A, 13-day-old WT and developmentally comparable 17-day-old *tic100^cue^^8^* mutant seedlings used to isolate chloroplasts for import assays. Seedlings were grown on 0.5% sucrose. Scale bar: 1 cm. B, Phosphor screen image of import reactions of *in vitro*-translated (IVT) RUBISCO SSU polypeptide, carried out with equal total numbers of chloroplasts isolated from the seedlings above. Samples were taken 2.5, 5, 7.5, or 10 min after the start of the reaction. The import reaction converts the precursor (pre.) into mature polypeptide (mat.) of reduced size. Results from one representative experiment. A Coomassie-stained total protein gel corresponding to the same experiment is also shown. C, Quantification of the amount of mature protein at each time point, normalized relative to the amount of mature protein in WT after 10 min of import. Average values from four independent experiments. Error bars represent sem. Values for *tic100^cue^^8^* were significantly different to those of WT at every time point (Student’s *t* test, *P* < 0.05).

Taking advantage of the opportunity afforded by the partial loss of function of TIC100 in *tic100^cue8^*, we carried out *in vitro* import assays with chloroplasts isolated from well-developed seedlings of the mutant, using a photosynthetic protein precursor, the Rubisco small subunit (SSU). Four independent experiments, using developmentally comparable WT and mutant plants (Figure 3A), revealed that *cue8* mutant chloroplasts import less than one-third of the amount of preprotein than the equivalent number of WT chloroplasts do (Figure 3, B and C).

To understand more clearly the basis for the protein import deficiency in the mutant, we analyzed the levels of several translocon components by immunoblotting. Equal amounts of total chloroplast proteins were loaded per lane. Band intensities for some translocon components, in both the outer (TOC75) and inner (TIC110, TIC40) envelope membranes, were elevated by about a third in the *tic100^cue8^* lanes (Supplemental Figure S4). This reflected the fact that *tic100^cue8^* chloroplasts are, to varying extents, less developed internally and contain reduced amounts of the major photosynthetic proteins, including Rubisco and LHCB (López-Juez et al., 1998), relative to WT, leading to the relative overloading of envelope components in the *tic100^cue8^* samples when using equal protein amounts (this also explains the slightly lower amounts of total protein in the *tic100^cue8^* samples following normalization according to equal chloroplast numbers in Figure 3B). Crucially, in spite of this, the level of TIC100 polypeptide was reduced to between one quarter and one-eighth that in the WT (Supplemental Figure S4) on an equal total chloroplast protein basis, or less than one-eighth when normalized to another envelope protein, TIC40. The decrease in TIC100 abundance in the mutant was linked to reductions in the levels of the other components of the 1 MDa complex (TIC20 and the additional TIC56 and TIC214; Supplemental Figure S4), to between 25% and 50% of WT levels when expressed relative to TIC40. These observations are consistent with the notion that these proteins associate, with the very substantial loss of TIC100 in the *tic100^cue8^* mutant preventing others from accumulating normally.

### Identification of a suppressor mutation of *tic100^cue8^*

**Figure 4:**
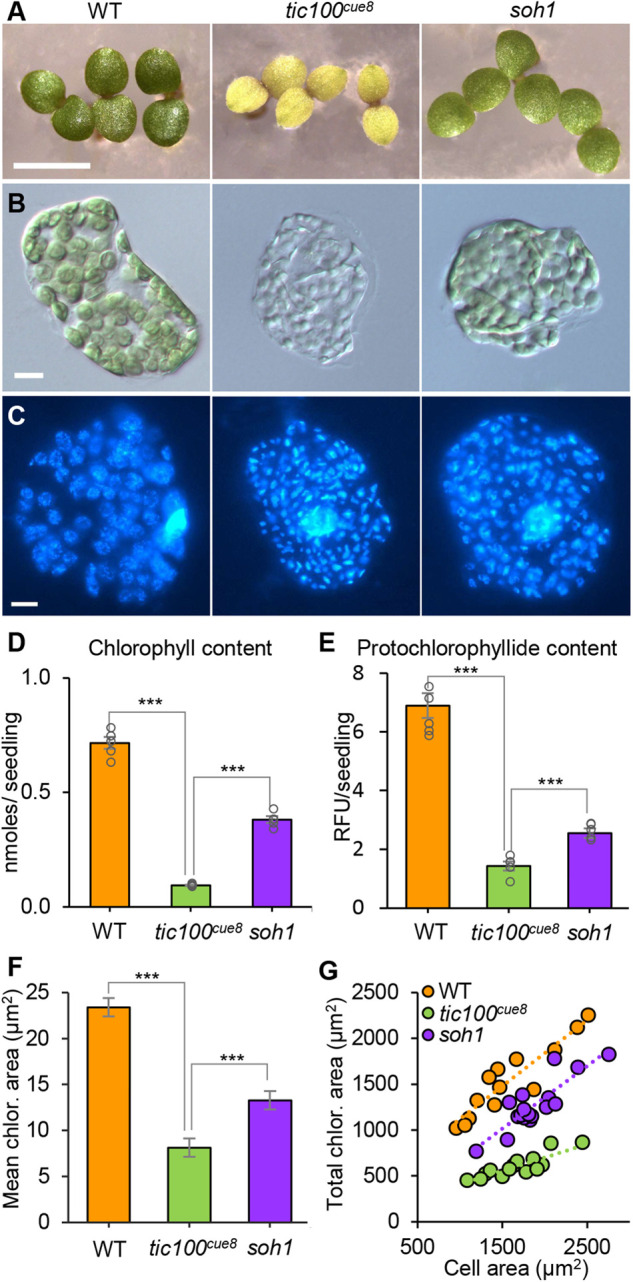
Identification of *soh1*, a suppressor mutant of *tic100^cue^^8^*, and phenotype of young cotyledon cells and their chloroplasts in WT, *tic100^cue^^8^* and *soh1* seedlings. A, Five-day (WT) and 6-day (*tic100^cue^^8^* and *soh1*) seedlings. Scale bar: 5 mm. B, Individual cells of the three genotypes, of seedlings equivalent to those in (A), observed under DIC microscopy, displaying the different degrees of cell occupancy by chloroplasts. C, Individual cells observed by fluorescence microscopy following DAPI-staining of double-stranded DNA, revealing both the nuclei and the presence and density of nucleoids in individual chloroplasts. Scale bar (B and C) 10 µm. D, Chlorophyll content per seedling for seedlings identical to those in (A). E, Protochlorophyllide content per seedling (relative fluorescence units) of 5-day-old seedlings of the three genotypes. F, Mean area of individual chloroplasts in cells equivalent to those in (B). G, Total plan area of chloroplasts in a cell plotted against cell plan area, for the three genotypes, including regression lines of best fit. The presented values are means and the error bars (in D and E) show sem from five biological replicates, each with at least five seedlings, or (in F) at least 10 individual chloroplasts from each of at least 13 individual cells total, obtained from at least four different cotyledons per genotype. For all panels, asterisks above lines denote comparisons indicated by the lines: ***P < 0.001 (Student’s *t* test).

A search for suppressor mutations of the *ppi1* mutant, defective in the TOC33 subunit of the outer envelope translocon, led to the identification of SP1, a ubiquitin ligase which remodels the import complexes to control protein import and plastid development (Ling et al., 2012, 2019). We sought to deepen our understanding of inner envelope translocation processes by searching for suppressors of the *tic100^cue8^* mutation. Screening of mutagenized M2 populations for increased levels of greening led to the identification of a mutant with a dramatic phenotype, which we named *suppressor of tic100 1*, *soh1* (Figure 4A). Backcrossing of *tic100^cue^^8^ soh1* into the *tic100^cue8^* parent resulted in 100% of the F1 (62 seedlings) showing a phenotype which was intermediate between that of the parents but closer to the *soh1* phenotype (Supplemental Figure S5A); while self-pollination of the F1 plants yielded 75% (554 out of 699, chi-squared *P* = 0.19) seedlings with suppressed phenotype, among which about a third displayed a marginally larger seedling phenotype (these plants represented a quarter of the total F2 population: 158 out of 699, chi-squared *P* = 0.21). These data indicated that *soh1* is a gain-of-function mutation that improves greening and growth, and which has a semi-dominant character.

We have recently shown that the virescent phenotype of *tic100^cue8^* is caused by early chloroplasts in very young cotyledons being small and unable to fill the available cellular space (Loudya et al., 2020). In this regard, chloroplasts of 6-day-old *soh1* seedlings were much more similar to those in the WT (Figure 4B). Consequently, plastid DNA nucleoids, tightly packed in *tic100^cue8^* as previously reported (Loudya et al., 2020), appeared much less dense in *soh1* (Figure 4C). Moreover, total chlorophyll of light-grown seedlings, protochlorophyllide of dark-grown seedlings, the average size of individual chloroplasts, and the mesophyll cellular occupancy by chloroplasts (the chloroplast index), which were all reduced dramatically in *tic100^cue8^*, were largely restored in *soh1* seedlings (Figure 4, D–G).

### Identification of the gene carrying the *soh1* mutation

**Figure 5:**
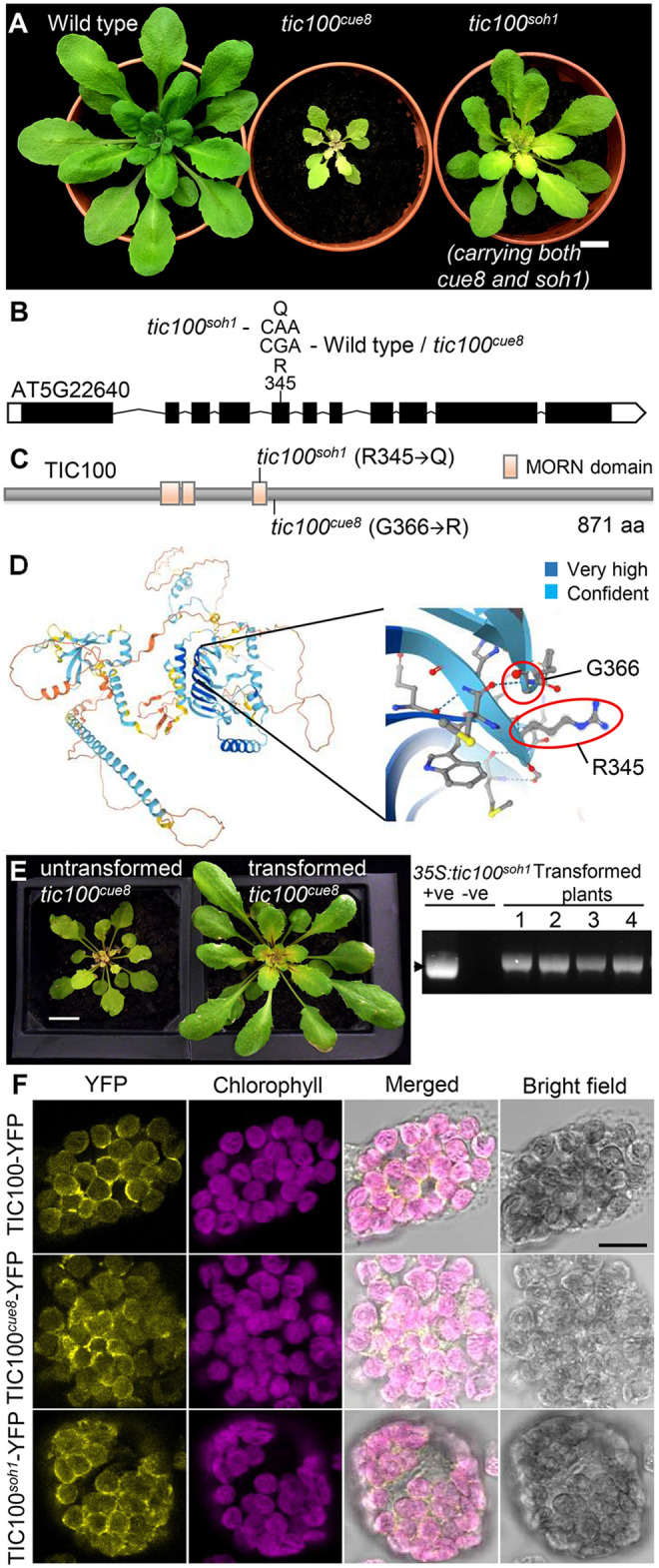
Cloning of the intragenic *suppressor of TIC100* (*soh1*) mutation, phenocopying and localization of TIC100. A, Soil grown plants of WT pOCA108, *tic100^cue^^8^* (*tic100^cue^^8^*) and suppressed *tic100^cue^^8^ soh1* double mutants (*tic100^soh1^*) are shown at 28 days of age. Scale bar: 1 cm. B, Second missense mutation (and resulting substituted amino acid position) in the genomic sequence of the *TIC100* gene present in the *tic100^soh1^* mutant and absent in *tic100^cue^^8^* or its WT parent. C, Model of the domain structure of the TIC100 protein, indicating the position of the single mutation present in *tic100^cue^^8^* or the two mutations present in the *tic100^soh1^* double mutant. The MORN domains occupy positions 219–239, 243–257, and 337–352. D, TIC100 protein structure prediction by Alphafold, showing the position of the WT amino acids affected by the *tic100^cue^^8^* and *tic100^soh1^* mutations. The legend indicates level of prediction confidence. E, Phenocopying of the suppressor *soh1* mutant by transformation of the single *tic100^cue^^8^* mutant with an over-expressed, double-mutated *tic100^soh1^* coding sequence (CDS) driven by the 35S promoter (as seen in 11 independent T1 plants, 4 shown). Plants shown at 30 days of age. Scale bar: 1 cm. Gel on the right confirms the genotype of the transformed plants. “+ve”: positive genotyping control (bacterial plasmid). F, Localization of the TIC100 protein, in its WT, TIC100^cue^^8^ and TIC100^soh1^ (double-mutated) forms, to the chloroplast periphery in transfected protoplasts. WT protoplasts were transfected with constructs encoding WT and mutant forms of TIC100, each one tagged with a C-terminal YFP tag. The protoplasts were analyzed by confocal microscopy, and representative images are shown. Scale bar: 10 µm.

We generated a mapping population by backcrossing the suppressor mutant as originally identified (*tic100^cue^^8^ soh1*, Bensheim ecotype) to the unmutagenized *tic100^cue8^* parent (also in the Bensheim ecotype). F2 seedlings of unsuppressed, *tic100^cue8^* phenotype were used for mapping by SHORT READS sequencing (SHOREmap) (Schneeberger et al., 2009), as described in Supplemental Figure S5, B and C. Mapping of the *soh1* mutation identified a region of chromosome 5 (Supplemental Figure S5D) spanning seven genes with mutations in the open reading frame, and one of these was *TIC100*. Sanger sequencing confirmed the presence in the *soh1* mutant of both the original *tic100^cue8^* mutation and a second mutation, which we provisionally named *tic100^soh1^* (Figure 5, A–C). Constitutive expression under the 35S promoter of a *tic100^cue^^8^ ^soh1^* cDNA carrying both of these mutations in the *tic100^cue8^* mutant plants resulted in T1 plants showing a suppressed phenotype (Figure 5E); the genotyping of these plants confirmed the presence of both *tic100^cue8^* (from the endogenous gene) and *tic100^cue^^8^ ^soh1^* alleles (from the transgene). In contrast, constitutive expression of the *tic100^cue8^* cDNA under the same promoter in *tic100^cue8^* plants produced only *tic100^cue8^* phenotypes (Supplemental Figure S6), demonstrating that it was the second mutation, and not overexpression of the gene, that caused the suppression effect. Therefore, we concluded that the second mutation was indeed responsible for the suppressed phenotype, and we hereafter refer to the *tic100^cue^^8^ ^soh1^* double mutant as *tic100^soh1^* (Figure 5A).

Analysis of the predicted domain structure of the TIC100 protein by searching in the Interpro domains database showed that the initial *tic100^cue8^* mutation occurred immediately outside the C-terminus of the third of three MORN domains, introducing a basic arginine residue in place of a neutral glycine. Conversely, the *tic100^soh1^* mutation replaced an arginine residue, within the third MORN domain (20 amino acids upstream of the *tic100^cue8^* substitution), with a neutral glutamine residue (Figure 5, B and C). 3D protein structure prediction by the recent, breakthrough AlphaFold algorithm (Jumper et al., 2021) indeed showed the amino acids affected by the two substitutions to lie in very close proximity in space, in regions of confidently predicted structure (Figure 5D), at one end of a large, highly confidently predicted β-sheet region which includes the MORN domains.

### TIC100^cue^^8^ and TIC100^soh1^ proteins retain localization at the chloroplast periphery

Previous biochemical analyses identified the TIC100 protein as part of the 1-MDa complex in the inner envelope membrane with a proposed role in preprotein import (Kikuchi et al., 2013; Chen and Li, 2017; Richardson et al., 2018). In view of the protein import defect of the mutant, described above, we asked whether the *tic100^cue8^* mutation interferes with the localization of the TIC100 protein, and whether such an effect might in turn be alleviated by the *tic100^soh1^* mutation. Therefore, we constructed YFP fusion versions of the TIC100 protein in its WT and two mutant forms (the second carrying both mutations). Transient overexpression of the fusions in protoplasts resulted in some accumulation of all three proteins in the cytosol of the cells (Supplemental Figure S7), which interfered with assessment of chloroplast envelope association. However, in protoplasts in which rupture of the plasma membrane eliminated the background cytosolic protein (which we interpret to be mislocalized owing to overexpression), TIC100 was clearly observed at the periphery of chloroplasts, possibly with a small amount of intra-organellar signal; this is consistent with the previous biochemically determined localization. Significantly, neither of the two mutations altered this character (Figure 5F), and so we concluded that the mutations affect a property of TIC100 other than its localization.

### *tic100^soh1^* corrects the protein import defect caused by *tic100^cue8^*

**Figure 6:**
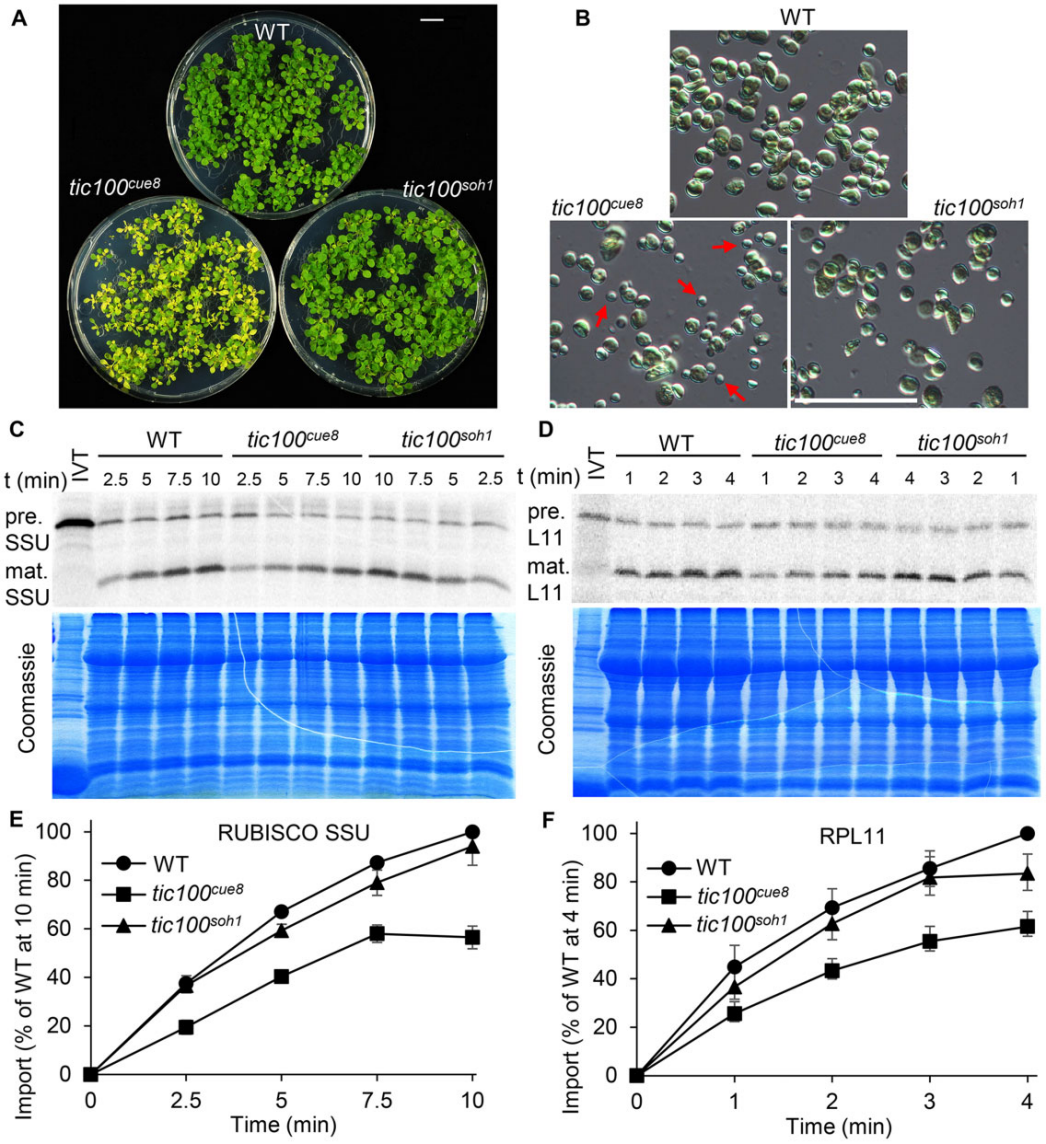
Chloroplasts of *tic100^cue^^8^* exhibit reduced import of a photosynthetic and a housekeeping preprotein, with different age-dependent import profiles, and both defects are suppressed by the second mutation in *tic100^soh1^*. A, 13-day-old WT and 17-day-old *tic100^cue^^8^* and *tic100^soh1^* mutant seedlings used to isolate chloroplasts for the import assays. Seedlings were grown on 1% sucrose. Scale bar: 1 cm. B, Examples of chloroplast populations used for the *in vitro* protein import assays. Occasional small chloroplasts in *tic100^cue^^8^* are indicated with arrows. Scale bar: 50 µm. C, Phosphor screen image of import reactions of IVT SSU polypeptide, carried out with equal numbers of *tic100^cue^^8^* and WT chloroplasts. Samples were taken at the indicated times after the start of the reaction. The import reaction converts the precursor (pre.) into the mature (mat.) polypeptide of reduced size. Results from one representative experiment are shown. The lower panel shows the corresponding Coomassie-stained total protein gel of the same experiment run separately. D, Import of RPL11 into equal numbers of WT, *tic100^cue^*^8^, and *tic100^soh1^* chloroplasts. Upper and lower panels as in (C). Values at every time point were significantly different for *tic100^cue^^8^* relative to WT, and for *tic100^soh1^* relative to *tic100^cue^^8^*. E, Quantitation of at least four independent protein import assays as that shown in (C), from four separate chloroplast populations obtained from at least four groups of independently grown plants. The presented values are means, and the error bars show sem. F, Quantitation of at least four independent import assays, as that shown in (D). Values at all time points for SSU and at 2, 3, and 4 min for RPL11 were significantly different for *tic100^cue^^8^* relative to WT, and for *tic100^soh1^* relative to *tic100^cue^^8^* (*P* < 0.05, Student’s *t* test).

Next, we asked whether the basis for the suppression of the *tic100^cue8^* phenotype in the *tic100^soh1^* mutant was a correction of the plastid protein import defect described earlier. In these experiments, the import of the photosynthetic SSU preprotein into equal numbers of chloroplasts isolated from WT, *tic100^cue8^* single-mutant, and *tic100^soh1^* double-mutant plants (Figure 6, A and B) was measured. On this occasion, the results demonstrated a reduction of protein import in *tic100^cue8^* to ∼55% of the WT level; the smaller reduction in import seen here, relative to Figure 3, was attributed to the slightly greater extent of development of the mutant plants on a higher sucrose concentration in the medium (Supplemental Figure S1). Notably, protein import into the suppressed mutant chloroplasts was restored almost completely to WT levels (over 90%) (Figure 6, C and E).

To further assess the role of TIC100 in relation to functionally different proteins, we additionally tested the import of the housekeeping plastid RPL11 protein (50S plastid ribosomal subunit protein 11). This also allowed us to assess whether reductions in import capacity in *tic100^cue8^* chloroplasts could simply be an indirect consequence of differences in the stage of development of mutant chloroplasts, since SSU and RPL11 have been shown to be preferentially imported by chloroplasts of younger or older leaves, respectively (Teng et al., 2012). To the contrary, we obtained very similar results for RPL11 to those we had obtained for SSU (Figure 6, D and F).

Overall, these protein import data (which were observed across four independent experiments per preprotein) revealed a clear import defect in *tic100^cue8^* for two proteins which display different developmental stage-associated import profiles (Teng et al., 2012), and which use different types of TOC complexes (Jarvis and López-Juez, 2013; Demarsy et al., 2014). This was consistent with the gene expression profile of *TIC100* (Supplemental Figure S3). Notably, this was also consistent with the fact that both SSU and RPL11 preproteins were previously shown to physically associate during import with components of the 1-MDa complex, including TIC100 (Kikuchi et al., 2018), Moreover, our results revealed a very pronounced correction of the import defects seen in *tic100^cue8^* chloroplasts in the *tic100^soh1^* suppressed mutant.

### *tic100^soh1^* restores levels of 1-MDa protein components in *tic100*^*cue8*^

**Figure 7:**
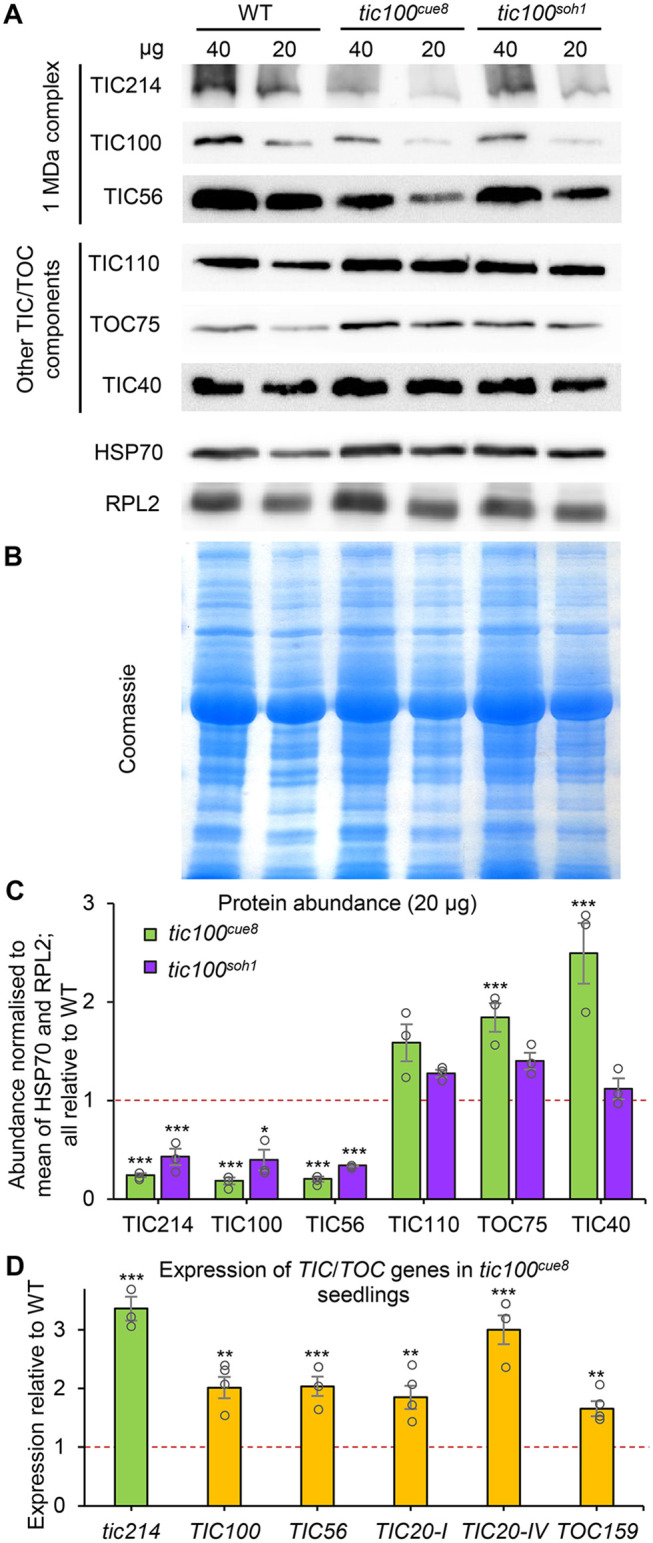
Chloroplasts of *tic100^cue^^8^* display decreased levels of 1-MDa complex proteins specifically, and this defect is suppressed by the second mutation in *tic100^soh1^*. A, Immunoblot analysis of total chloroplast proteins from preparations from WT (13-day-old), and *tic100^cue^^8^* and *tic100^soh1^* (17-day-old), seedlings (see Figure 6). The amount of protein (µg) loaded is indicated above each lane. The antibodies used for the detection of components of the 1-MDa complex (TIC56, TIC100, and TIC214) or other chloroplast envelope proteins (TOC75, TIC40, and TIC110) are indicated. Note the reduced amounts of components of the 1-MDa complex, which is apparent despite the increased loading of envelope proteins (as revealed by the levels of other polypeptides) specifically in the *tic100^cue^^8^* samples. Very limited antibody availability precluded probing the chloroplast protein extracts for the levels of TIC20. B, Coomassie-stained total protein gel of the same experiment. C, Quantitation of protein abundance from an analysis of the 20-µg samples in three independent experiments, relative to the mean of HSP70 and RPL2 in each sample, all expressed relative to WT protein levels. The presented values are means, and the error bars show sem. Asterisks represent significance of difference of each mutant relative to WT (Dunnett’s test). D, Expression, measured by reverse transcription-quantitative PCR, of *TIC*/*TOC* genes in *tic100^cue^^8^* seedlings similar to those analyzed in Figure 3, measured relative to expression in WT seedlings. Note *tic214* is chloroplast-encoded. The presented values are means, and the error bars show sem. of three RNA samples (biological replicates), each with two technical replicates. Asterisks represent significance of difference between mutant and WT: *P < 0.05, **P < 0.01, ***P < 0.001 (Student’s *t* test). Dotted lines represent protein levels (C) or expression (D) in WT.

Given the strong reductions in levels of 1-MDa TIC complex components (but not of other inner or outer envelope proteins) seen in *tic100^cue8^* mutant chloroplasts (Supplemental Figure S4), we asked whether the *tic100^soh1^* mutation had corrected the accumulation of components of the 1-MDa complex. Immunoblot analyses indicated that this was indeed the case (Figure 7, A–C). Analysis of TIC100 protein using the same chloroplast preparations as used for import assays in Figure 6 showed partial restoration of the level of this protein in the double mutant. Furthermore, qualitatively similar trends to those seen for TIC100 were observed for TIC56 and TIC214, but we were unable to quantify TIC20 here due to very limited availability of the corresponding antibody. In contrast, no such protein level reduction in *tic100^cue8^*, or restoration in *tic100^soh1^*, was observed for control housekeeping, non-membrane proteins (HSP70 and RPL2); in fact envelope proteins unrelated to the 1-MDa complex (TIC110, TIC40, and TOC75) appeared elevated in *tic100^cue8^*, consistent with an enrichment of envelope proteins per unit total chloroplast protein, as discussed earlier, and accordingly returned to normal apparent levels in *tic100^soh1^*.

It could be argued that the reduced accumulation of the 1-MDa TIC might have been an indirect result from an effect of the *tic100^cue8^* mutation *via* retrograde signaling, leading to reduced nuclear gene expression and synthesis of translocon components. Such an explanation is highly unlikely given that we have previously observed elevated, not reduced, expression of nuclear and chloroplast-encoded genes for early-expressed, plastid housekeeping proteins in the mutant (Loudya et al., 2020). This is part of its “juvenile plastid” phenotype. In fact, we confirmed here that the expression of nucleus-encoded genes for 1-MDa TIC components and, especially, of the plastid-encoded *tic214* gene, were all elevated in *tic100^cue8^* (Figure 7D); the expression of the control *TOC159* gene was also elevated. Interestingly, and as previously observed for other elements of the juvenile plastid phenotype (Loudya et al., 2020), a substantial component of the retro-anterograde correction did not involve GUN1 action, since it occurred in *tic100^cue8^* even in the absence of GUN1 (Supplemental Figure S8).

Moreover, the transcript levels of *TIC20-IV*, which encodes an alternative form of TIC20 that functions independently of the 1-MDa complex (Kikuchi et al., 2013), were also elevated in *tic100^cue8^* (Figure 7D). Thus, we conclude that accumulation of subunits of the TIC 1-MDa complex is reduced by the *tic100^cue8^* mutation, that this occurs in spite of the attempted “retro-anterograde correction” at the gene expression level brought about by retrograde signaling, and that the accumulation of TIC 1-MDa complex subunits is partially restored by the *tic100^soh1^* mutation. Furthermore, a potential compensatory effect of the *tic100^cue8^* mutation, increasing the expression of *TIC20-IV* encoding an alternative TIC20 form that acts independently of the 1-MDa complex, is apparent.

### Interplay between *tic100* mutations and retrograde signaling

**Figure 8:**
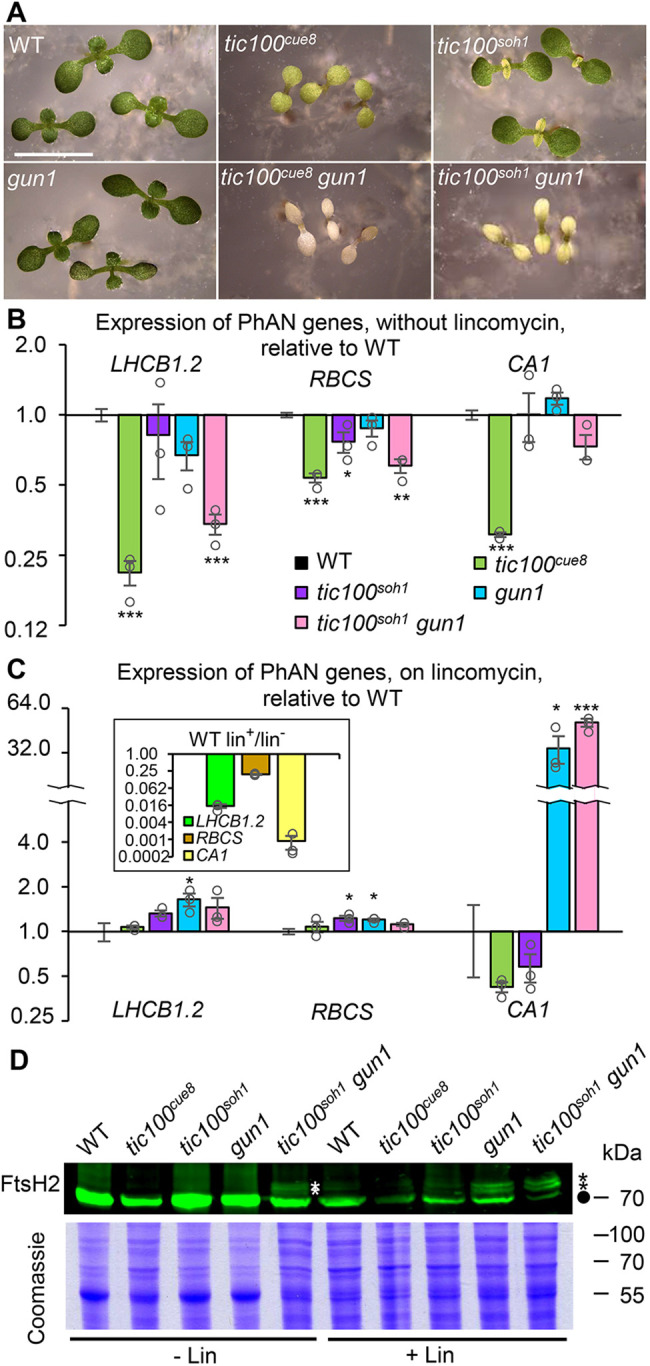
The *tic100^soh1^* mutation suppresses the synthetic seedling lethality of *tic100^cue^^8^* in combination with *gun1*, while *gun1* retains its “uncoupled” phenotype, and unimported FtsH2 precursors can be detected in the *tic100* mutants. A, Phenotype of 7-day-old seedlings of the genotypes indicated. *tic100^cue^^8^ gun1* double mutants exhibit eventual seedling mortality. Scale bar: 5 mm. B and C, Photosynthesis-associated nuclear gene (PhANG) expression in the absence (B) or presence (C) of lincomycin in the genotypes indicated. Asterisks denote significance of difference between mutant and WT as indicated for Figure 7. The inset compares the phenotype of the WT grown on lincomycin with that in its absence. Differences for all three genes exhibited *P* < 0.001. D, Immunoblot detection of high molecular weight (HMW) bands, previously shown to represent unimported, cytosolic precursors (asterisks) of chloroplastic FtsH2 (circle), in the presence or absence of lincomycin, in the genotypes indicated. About 80 µg of protein were loaded per lane. Equal loading shown by Coomassie Brilliant Blue total protein staining (10 µg of protein per lane).

We previously observed that while the single *tic100^cue8^* mutation resulted in virescence, the simultaneous loss of GUN1 (which in itself causes partial uncoupling of nuclear gene expression from the state of the plastid) was incompatible with survival—that is, combination of the *tic100^cue8^* and *gun1* mutations resulted in synthetic seedling lethality in the double mutants (Loudya et al., 2020). To further investigate the extent of suppression in *tic100^soh1^*, we analyzed *tic100^soh1^ gun1* triple mutants. In contrast to the *tic100^cue^^8^ gun1* albino, seedling-lethal phenotype, the *tic100^soh1^ gun1* triple mutations caused plants to be very pale but were not lethal (Figure 8A), and indeed the plants could survive and produce seeds entirely photoautotrophycally under low light conditions. In other words, the synthetic lethality of *tic100^cue^^8^ gun1* double mutations was suppressed by *tic100^soh1^*.

Evidence has recently accumulated for a role of altered organelle proteostasis in chloroplast retrograde signaling (Tadini et al., 2016; Wu et al., 2019; Tadini et al., 2020). The clear virescence exhibited by *tic100^soh1^*, and its strong, albeit reduced, genetic interaction with loss of GUN1, led us to ask whether retrograde signaling was in any way altered by impairment or recovery of TIC100 function. Two scenarios were in principle possible: In the first, a strong response of reduced photosynthesis-associated nuclear gene (PhANG) expression might be observed when TIC100 function is reduced, resulting in the virescence of *tic100^cue8^* and even *tic100^soh1^* mutants, and the very low PhANG expression in *tic100^cue8^* (Vinti et al., 2005). In this scenario, GUN1 function, mediating retrograde signaling, would remain fundamentally unchanged in the mutants. In the second scenario, the impairment of protein import occurring in *tic100^cue8^*, but barely so in *tic100^soh^*^1^, would result in the accumulation of unimported proteins in the cytosol of the mutants, and this in turn, as proposed by Wu et al. (2019), would itself cause elevated PhANG expression in spite of the chloroplast damage, that is, a *genomes uncoupled* (*gun*) phenotype. We examined these two possible, contrasting scenarios by quantifying transcript levels of *LHCB1.2*, *RBCS*, and *CA1*—the first two genes classically monitored in retrograde analyses, and the third gene showing one of the greatest extents of reduction by treatment with lincomycin (Koussevitzky et al., 2007)—in the presence of a chloroplast translation inhibitor which triggers a dramatic loss of PhANG expression. We exposed to lincomycin seedlings of each *tic100* mutant, and of the *tic100^soh1^ gun1* triple mutant. We did not examine *tic100^cue^^8^ gun1*, as such albino seedlings become impossible to select in the presence of the antibiotic from the segregating population in which they occur. The results of this analysis (Figure 8, B and C) are clearly consistent with the first scenario: reductions in PhANG expression were strong in the absence of lincomycin in *tic100^cue8^* and in the *tic100^soh1^ gun1* triple mutant (Figure 8B)—as originally seen in the *tic100^cue^^8^ gun1* double (Loudya et al., 2020)—and mild in *tic100^soh1^*. The reductions in the triple mutant were not due to the *gun1* mutation having lost its associated “uncoupled” phenotype, but rather to the strong chloroplast defect (manifested as a greening defect) it suffered. This was shown by the fact that in *tic100^soh1^ gun1* PhANG expression, particularly that of *CA1*, was clearly uncoupled, that is, much less reduced by lincomycin than it was in the WT (Figure 8C). We conclude that, as anticipated, *tic100^cue8^* is not a *gun* mutant. We also conclude that the capacity of GUN1 to initiate retrograde communication remains strong in *tic100^soh1^*, and that it can therefore explain the retained, pronounced virescence.

PhANG expression changes, in particular in seedlings grown on lincomycin, have been attributed to the presence of unimported precursor proteins, which can be detected in whole-cell and cytosolic extracts of such antibiotic-treated plants (Wu et al., 2019; Tadini et al., 2020). Such precursors have also been detected in *gun1* even in the absence of antibiotic (Tadini et al., 2020), and one could speculate that they might be also detectable in the *tic100* mutants. We examined this through immunoblot analysis of the FtsH2 protein, a thylakoid-associated chaperone for which high molecular weight (HMW) bands have been previously observed (Tadini et al., 2020) using the same antibody. The HMW bands were previously shown to represent cytosolic, unimported precursors by cellular fractionation and by the construction and examination of FtsH2-GFP fusion proteins. Our results (Figure 8D) confirmed that the level of mature FtsH2 protein is much reduced in extracts of lincomycin-treated seedlings. Furthermore, the antibody could detect the presence of the same HMW bands (unimported protein) in extracts of seedlings grown in the presence of the antibiotic, and also in those of *tic100^soh1^ gun1* seedlings in its absence, an observation which is consistent with a chloroplast protein import defect in that case. They were, however, seen in *tic100^cue8^* only in the presence of the antibiotic.

### Editing of chloroplast mRNA is altered in *tic100^soh1^ gun1* seedlings in a manner consistent with the juvenile plastid phenotype

**Figure 9:**
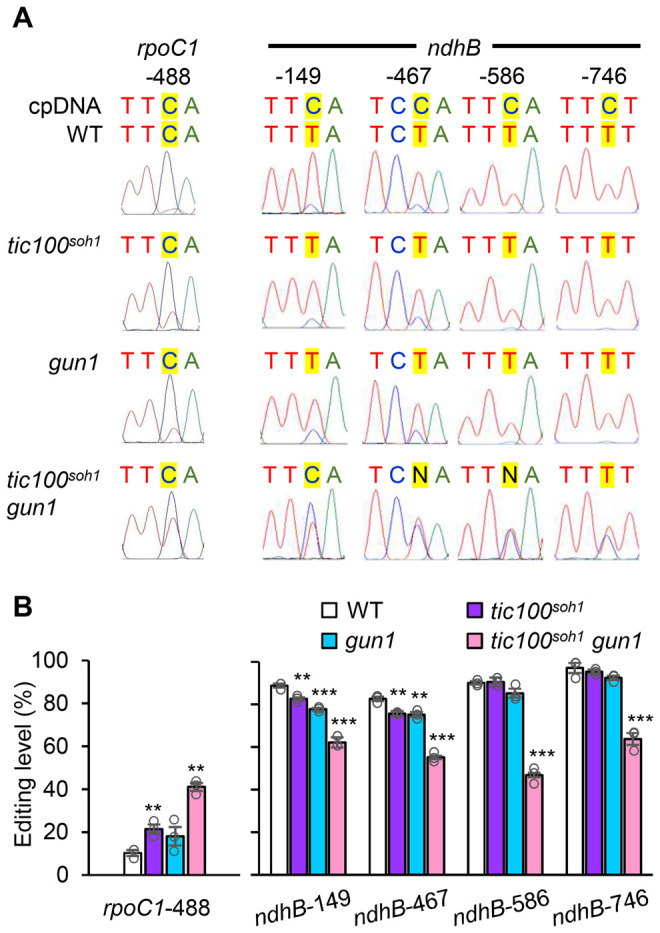
Editing of chloroplast mRNA is increased for the transcripts of *rpoC1* and decreased for those of *ndhB* in *tic100^soh1^ gun1* seedlings. A, Representative sequence electropherograms of cDNA generated from RNA of the genotypes indicated. The original, genomic chloroplast (cp) DNA sequence is indicated at the top. B, Quantitation of the degree of editing of the two plastid mRNAs shown in (A), averaged for three independent cDNA preparations from different seedling samples per genotype. Asterisks denote significance of difference between mutant and WT as indicated in Figure 7.

RNA editing occurs for many chloroplast transcripts and it has been previously demonstrated that growth of seedlings on norflurazon (which blocks carotenoid synthesis) or lincomycin, and the concomitant disruption of chloroplast development, results in changes in the extent of editing of different chloroplast transcripts (Kakizaki et al., 2012). Importantly, such changes, involving both increases and decreases in editing, were also observed in plants defective in the TOC159 outer membrane translocon receptor (Kakizaki et al., 2012). They were also seen in *tic100^cue8^* and, even more dramatically, *tic100^cue^^8^ gun1* (Loudya et al., 2020) and they involved increased editing of *rpoC1*, encoding a subunit of the chloroplast RNA polymerase, and decreased of *ndhB*, encoding a photosynthetic electron transport protein. We interpreted such changes, not as evidence of a direct role of CUE8 (TIC100) in editing, but as part of the “juvenile plastid” phenotype of the mutants. That interpretation is consistent with the existence of two phases of organelle biogenesis: an early, “plastid development” phase (photosynthesis-enabling but pre-photosynthetic, involving expression of the chloroplast genetic machinery), and a later, “chloroplast development” phase involving photosynthetic gene expression (as seen particularly clearly in developing cereal leaves) (Chotewutmontri and Barkan, 2016; Loudya et al., 2021). We asked whether this aspect of the *tic100^cue8^* phenotype had also been suppressed by *tic100^soh1^*. This was indeed the case (Figure 9): the extent of editing of *rpoC1* and *ndhB* transcripts was indistinguishable between *tic100^soh1^* and the WT (or *gun1*). In contrast, editing was increased for *rpoC1*, and reduced for *ndhB*, in the *tic100^soh1^ gun1* double mutant, which we again interpret as a more juvenile state of chloroplast development caused by the combination of the mild loss of import capacity and the simultaneous loss of GUN1 function.

## Discussion

### Genetic evidence supports the broad involvement of TIC100 in protein import

The nature of the proteins imported into plastids clearly determines plastid type and functions, and the identity of the import receptors even influences whole-plant performance in the face of stress. This underscores the importance of questions concerning the exact composition of the protein translocons. Indeed, the identity of the inner membrane TIC machinery has been the subject of much debate, particularly concerning the identity of the channel and the import motor (de Vries et al., 2015; Nakai, 2015b; Bolter and Soll, 2017; Sjuts et al., 2017; Schafer et al., 2019; Nakai, 2020; Richardson and Schnell, 2020). The serendipitous identification of *cue8* as a hypomorphic allele of *TIC100* provided an opportunity to begin addressing one such area of controversy. Knockout mutants of *TIC100*/*EMB1211* (a component of TIC 1-MDa complex) suffer from severe embryo development defects, leading to very early seedling lethality (Liang et al., 2010; Kikuchi et al., 2013). Attempts have previously been made to address the role of the 1-MDa complex by assessing the import characteristics of seedlings harboring the seedling-lethal knockout mutation *tic56*-1, by proteomically analyzing chloroplast-targeted proteins (Kohler et al., 2015). However such analysis is indirect and cannot, by definition, reveal differences in import rate, since seedlings are examined after lengthy *in vitro* culture. A second mutant, *tic56*-3, expresses a truncated TIC56 protein and has a mild phenotype, allowing the execution of *in vitro* assays to determine chloroplast protein import rates (Kikuchi et al., 2013; Kohler et al., 2015). Perhaps owing to different growth or chloroplast preparation conditions, those experiments did (Kikuchi et al., 2013) or did not (Kohler et al., 2015) observe reduced import rates in the mutant. In fact, one analysis reported very low levels of 1-MDa complex subunits overall in the *tic56*-3 mutant, and failed to detect TIC100 at all (Schafer et al., 2019), while in the same mutant TIC100 had previously been readily observable (Kikuchi et al., 2013). Understandably, it has been difficult to reach consensus based on data obtained from such genotypes.

An alternative approach may help to provide a resolution. Informative *in vitro* protein import rate assays were readily performed in the current study using the chloroplasts of *tic100^cue8^*, which are severely impaired in TIC100 accumulation due to the *tic100^cue8^* mutation. Our data demonstrated a clear reduction in the efficiency of protein import into mutant chloroplasts, which cannot be explained simply by their reduced size or extent of development. Importantly, we have previously observed a physical and gene expression phenotype consistent with a juvenile state of plastid development in *tic100^cue8^* plastids (Loudya et al., 2020), and it is well known that plastids of young plants generally achieve greater, not inferior, import efficiencies (Dahlin and Cline, 1991). The use of two different preproteins with different age-dependency of import profiles (Teng et al., 2012; Chu et al., 2020), both of which exhibited reduced import in *tic100^cue8^*, indicated that the import reduction was not an indirect consequence of an altered developmental stage of the mutant organelles. On the contrary, the reduced protein import seen in *tic100^cue8^* chloroplasts provided a clear molecular explanation for the mutant’s strongly virescent phenotype, while the increased rate of protein import seen in *tic100^soh1^* chloroplasts explained the reduction of this virescence. Our results also confirm the need for functional TIC100 for the normal import of different types of preproteins, exemplified by Rubisco SSU, a model photosynthetic protein, and RPL11, a nonphotosynthetic protein.

### Genetic evidence supports a role for the 1-MDa complex in protein import

The link between the abundance of TIC100 and that of its putative 1-MDa complex partners, in both *tic100^cue8^* and the intragenic suppressor mutant *tic100^soh1^*, is consistent with the existence of this complex. Furthermore, the protein accumulation profile data revealed a correlation between protein import rates and 1-MDa complex protein levels: decreased (*tic100^cue8^*) or increased (*tic100^soh1^*) protein import rates were observed as subunits of the complex decreased (*tic100^cue8^*) or increased (*tic100^soh1^*), in a genetically-determined manner. The data further showed that some loss of the subunits, as seen in *tic100^soh1^*, can be tolerated with minimal deleterious effect on protein import at the chloroplast-containing seedling stage; an equivalent phenomenon could potentially explain some of the previous conflicting observations on *tic56*-3. Thus, our data are consistent with the existence of the TIC100-containing 1-MDa complex and with it having a role in protein import. Nonetheless, we emphasize that the data presented here do not in any way rule out an important role for TIC110 in protein import; for example, this protein may function at a later stage in the import process, by providing a scaffold for the coordination of stromal chaperones as previously proposed (Inaba et al., 2003; Jarvis and López-Juez, 2013; Tsai et al., 2013; Richardson and Schnell, 2020).

Among the difficulties raised concerning the proposed role of the 1-MDa complex in protein import, one is of a phylogenetic nature: the absence of several of the complex’s main components from the grass family of monocots (de Vries et al., 2015; Bolter and Soll, 2016). In contrast, recent evidence strongly supports the function of the complex in a chlorophyte alga (Ramundo et al., 2020). It has been argued that an alternative form of the TIC20 translocon may operate in grasses and that this utilizes orthologs of TIC20-IV which is expressed and presumably active in roots of Arabidopsis (Kasmati et al., 2011; Nakai, 2015a). In this light, it is worth noting that the developmental impairment seen in roots of *tic100^cue8^* was not as pronounced as that observed in shoot meristem-derived tissues, suggesting a reduced need for TIC100 in the roots compared to shoots. Interestingly, we observed in *tic100^cue8^* a three-fold increase in expression of *TIC20-IV*. This may have resulted in some compensation for the loss of function of TIC20-I that occurs in the absence of its 1-MDa partners, as the level of expression of *TIC20-I* is four-fold higher than that of *TIC20-IV* in emerging seedling cotyledons and six-fold higher in young leaves (Klepikova et al., 2016). TIC20-IV, independent of the 1-MDa complex, might have also escaped the obvious post-transcriptional regulation we observed for components of the complex in the *tic100^cue8^* mutant. Nevertheless, we do not believe that TIC20-IV could fully compensate for defects in 1-MDa complex, given the lethality of complex knockout mutations. It is apparent that there are some unquestionable differences between grasses and the majority of other flowering plant groups in terms of chloroplast biogenesis in leaves. For example, while the greening of dicot leaf primordia is noticeable from the very youngest stages, the greening of grass leaves occurs over a longer developmental period. Differences have also been seen for otherwise important components involved in organelle biogenesis; for example, grass family genomes contain genes for one chloroplast-targeted and one mitochondrion-targeted RNA polymerase, but no gene for a protein targeted to both, whereas dicots do carry such a dual-targeted enzyme (Borner et al., 2015).

At present, we can only speculate on the specific role of TIC100 within the 1-MDa complex. The nature of the suppressor mutation present in *tic100^soh1^* highlighted the importance of at least one of the three recognized MORN domains in the protein. Such domains are important in other proteins for membrane association and for association with specific lipids (Takeshima et al., 2000). We should stress that the suppressor mutation is unlikely to, in itself, have a positive effect on TIC100 function. It rather removes a native positive charge present in very close proximity to the additional positive charge introduced by the *tic100^cue8^* mutation. It is therefore likely to have removed an electrostatic repulsion in the TIC100^cue8^ mutant protein, which allowed the conformation of TIC100 to return to a mildly impaired state, close to its native one. Previous experiments (Kikuchi et al., 2013) have revealed that this protein, like TIC56, most likely occupies an intermembrane space position associated with the inner envelope membrane, while TIC20 (the protein with channel properties; Kovacs-Bogdan et al., 2011) and TIC214 are integral membrane proteins. Our study also confirmed a localization consistent with envelope association for TIC100, and one may speculate that this association is partly mediated by the MORN domains. However, our confocal microscopy analysis did not reveal any change in localization in the TIC100^cue8^ and TIC100^soh1^ mutant proteins. Therefore, we interpret that the mutations either affect or rescue some other aspect of TIC100 function, such as interactions with other members of the 1-MDa complex to promote complex stability.

A role (possibly an additional one) for TIC56 in chloroplast ribosome assembly has been reported, and mild translation inhibition phenocopies many aspects of the *tic56*-3 phenotype (Kohler et al., 2016). These observations have been raised as an objection against the involvement of the TIC56 protein, and by extension of the 1-MDa complex, in protein import. However, intriguingly, such translation inhibition also phenocopies the phenotype caused by the loss the receptor protein TOC159, which has an extremely well established role in import (Kohler et al., 2016). Reduced accumulation upon translation inhibition of two import-related but chloroplast-encoded proteins, TIC214 and Ycf2/FtsHi—the latter a subunit of a putative import motor (Kikuchi et al., 2018)—might explain such similarities.

### Chloroplast protein import is a source of plastid-to-nucleus communication which, through virescence, has adaptive value

Intriguingly, *tic100^soh1^* exhibited almost complete rescue of protein import rates and of greening in fully developed leaf tissue, and yet it still displayed a pronounced early virescence phenotype. This virescence is consistent with a strong early influence on plastid-to-nucleus communication (a strong early retrograde signal, should the signal be a negative regulator of photosynthetic gene expression; or a strong absence of one, should the signal be a positive regulator). Indeed, the simultaneous occurrence of *tic100^soh1^* and *gun1* mutations caused very severe greening deficiency, but not seedling lethality. Combined loss of TOC159 and GUN1 has also been reported to have severe consequences (in this case, seedling lethality) (Kakizaki et al., 2012). A number of important connections between GUN1 and the chloroplast protein import apparatus have recently been uncovered. *gun1* mutants lose subunits of the import translocons, including TIC100, in response to mild inhibition of chloroplast translation, to a greater extent than the WT does (Tadini et al., 2020). A 50% reduction in levels of import translocon subunits in *gun1*, observed on antibiotic-free medium (Tadini et al., 2020), could have made seedlings somewhat more sensitive to the defects brought about by the *tic100* mutations. However, that cannot fully explain the very strong genetic interactions between the respective mutations, reaching seedling lethality in the case of *tic100^cue^^8^ gun1*. *gun1* also causes mild but synergistic decreases in import rates in a mutant defective in chloroplast proteostasis, and GUN1 physically associates with chloroplast chaperones that act in protein import (Wu et al., 2019). Both of those studies (Wu et al., 2019; Tadini et al., 2020) demonstrated the accumulation of unimported preproteins in the WT in the presence of lincomycin, and in *gun1* even in its absence. We observed HMW bands of FtsH2 that likely represent such unimported preprotein, and could detect such bands in young seedlings grown on lincomycin and of *tic100^soh1^ gun1* seedlings even in the absence of lincomycin. Wu et al. (2019) also observed the emergence of a cellular folding stress response in the cytosol of chloroplast proteostasis mutants, consistent with the presence of such unimported proteins. We should note, though, that our evidence is consistent with the import defects and potential accumulation of unimported preproteins in the cytosol playing a major signaling role which results in the reduction, not the maintenance, of PhANG expression. The reduction in PhANG expression in response to impairment of TIC100 function particularly in *tic100^cue8^*, or to exposure to lincomycin, both of which may or do lead to import defect or preprotein accumulation, is consistent only with such a negative role. Therefore, our data do not support this aspect of the previously proposed model (Wu et al., 2019), of a role for increased accumulation of preproteins in itself in elevating PhANG expression and therefore being the cause of the *gun* phenotype in the *gun1* mutant. We also did not observe a *gun* phenotype in *tic100^cue^*^8^, nor, in fact, was a *gun* phenotype observed in *toc33* (*ppi1*), *toc75-III*-3, and *tic40*-4 mutants (Wu et al., 2019). Our data support a loss of protein import at the inner envelope bringing about a reduction in PhANG expression and triggering a retro-anterograde delay in chloroplast development which requires GUN1 and, by allowing gradual correction of the defect, has adaptive value (Loudya et al., 2020). Our earlier and current data on RNA editing in *tic100* mutants also support the notion that the shifts in degree of RNA editing occurring for different chloroplast transcripts also constitute part of such a retro-anterograde correction.

Taking these observations together, it is becoming apparent that the status of organelle protein import—particularly at the inner envelope membrane, mediated by the 1-MDa TIC complex—and protein homeostasis are critically interlinked with intracellular communication, and monitoring them is a critical function of chloroplast retrograde signaling, and of the GUN1 protein specifically. According to our observations, and consistently with previous ones (Kubis et al., 2003), impaired protein import reduces PhANG expression. How GUN1 relays information of changes in import status appears to remain unresolved and warrants future exploration.

## Materials and methods

### Plant material and growth conditions

The *A.* *thaliana cue8* mutant (López-Juez et al., 1998; Vinti et al., 2005) and its WT pOCA108, in the Bensheim ecotype, have been described. The *gun1*-*1* mutant, in the Col-0 ecotype, was previously described (Susek et al., 1993). The *cue8* and *soh1* mutations were backcrossed into Col-0 for double mutant analysis as described (Loudya et al., 2020). The generation of the *soh1* mutant is described below. Plants were grown in soil under 16-h photoperiods and a fluence rate of 180 µmol m^−2^ s^−1^ (TLD 840; Sylvania, Newhaven, UK), and seedlings grown *in vitro* in MS media supplemented with 1% sucrose, unless otherwise indicated (Supplemental Figure S1) under continuous white light, at a fluence rate of 100 µmol m^−2^ s^−1^, as previously described (López-Juez et al., 1998; Loudya et al., 2020). Genotyping of the individual mutations (following gene identification), individually or for double mutant generation, used PCR followed by restriction digestion (Supplemental Table S4).

### Analysis of plastid development

WT and *cue8* lines carrying the DsRed reporter gene targeted to chloroplasts (Haswell and Meyerowitz, 2006) were identified following a cross and selected to homozygosity. Cotyledons and roots from *in vitro*-grown seedlings (7-day-old) were mounted on slides and observed using a Nikon (Kingston upon Thames, UK) Eclipse NI fluorescence microscope, ×20 Plan Fluor objective and Texas Red filter block. Cotyledons of non-DsRed, negative control seedlings were examined to confirm that the majority of the fluorescence signal was attributable to the DsRed plastid reporter (Figure 1). Fluorescence images of the same type of tissue used identical exposure conditions.

Five-day-old WT and 6-day-old mutant seedlings were fixed (Figure 4) in 3.5% glutaraldehyde and subject to cell separation in 0.1 M EDTA, pH 9, prior to observation in a differential interference contrast Nikon Optiphot-2 microscope. Cells (*n* = 13–18) of four independent cotyledons per genotype were observed, with cell plan areas, chloroplast number and individual area calculated as described (Loudya et al., 2020).

### Analysis of root development

Seedlings were cultured *in vitro*, under the conditions described in Supplemental Figure S1, images taken and root length quantified using ImageJ (ImageJ.net) software.

### Map-based cloning of *cue8*

Two mapping populations were generated following a *cue8* × La-*er* cross. In one, F2 mutant plants were selected (genotype *cue8*/*cue8*); in another, WT plants were selected, grown to maturity and their progeny individually scored to identify plants without *cue8* progeny (genotype *+*/*+*). A third mutant mapping population was generated following a *cue8* x Col-0 cross. In total, 344, 557, and 619 plants were selected respectively (total 1,520 plants) in the three mapping populations. Plants were examined at polymorphic markers 541 and 692 (Col-0 population) or 576 and 613 (La-*er* populations). DNA was extracted from pools of three to four plants, was examined and, if a recombination event identified, individual plants were retested to identify the recombinant. Other polymorphisms between Col-0 and La-*er* (TAIR, www.arabidopsis.org) were developed as polymorphic markers by designing flanking primers for PCR amplification and differential enzyme digestion (Supplemental Table S1), and screening Col-0, La-*er*, and pOCA108 genomic DNA. pOCA108 sequence was more frequently found to be polymorphic against La-*er* (13/20) than against Col-0 (7/20). Genes in the region of interest were ruled out by isolation of KOs of the SALK collection following genotyping with the respective forward, reverse, and border primers (Alonso et al., 2003; Supplemental Table S2). When no homozygous KO was identified (AT5G22600, AT5G22640, AT5G22650, AT5G22660, AT5G22670, AT5G22680, AT5G22710, and AT5G22730), primer pairs were designed covering the full open-reading frame, and amplicons obtained using *cue8* genomic DNA template submitted for Sanger sequencing (DNASeq, Medical Sciences Institute, University of Dundee, UK). When polymorphisms against the TAIR sequence occurred, amplicons for the pOCA108 WT were also sequenced (AT5G22640, AT5G22650, AT5G22660, AT5G22670, AT5G22710, and AT5G22730) to compare.

### Vector construction and complementation

A TAC (Liu et al., 2000), JatY-57L07, containing the genomic region covering genes AT5G22640 to AT5G22740, was obtained as an *Escherichia* *coli* stab culture from the John Innes Centre (Norwich, UK). The TAC was introduced into *Agrobacterium* strain GV3101 by electroporation, followed by transformation of Arabidopsis *cue8* using the floral dip method (Clough and Bent, 1998). Selection of transformants utilized resistance to BASTA (Glufosinate), sprayed at 150 mg/L as a mist every 3 days. Diagnostic PCR (for primers see Supplemental Table S7) produced a 607-bp amplicon.

To produce a full-length WT cDNA, RNA was isolated (RNeasy kit, Qiagen, Manchester, UK) from pOCA108 plants, cDNA synthesized (AMV Reverse Transcriptase kit, Promega, Southampton, UK) and amplified (Supplemental Table S7) using BIO-X-ACT Long DNA polymerase (Bioline, London, UK). The 2,622-bp product was directionally cloned by ligation into the pENTR/D-TOPO vector (Invitrogen/Thermo Fisher Scientific, Hemel Hempstead, UK). Digestion with EcoRV and NotI generated 2,742 and 2,435 bands, confirming the cloning of the full-length cDNA, and that with EcoRI and EcoRV generated 591 and 4,586 bp bands, confirming the forward orientation. Sequencing confirmed the absence of errors. The TOPO vector insert was cloned into pB7WG2 vector (Karimi et al., 2002) using Gateway recombination, to produce the pB7WG2/CUE8c construct. The pB7WG2 vector includes an upstream 35S promoter. Sequencing confirmed the correct orientation and absence of errors. Transformation of pB7WG2/CUE8c used the floral dip method. Transformants were selected using BASTA. Diagnostic PCR (Supplemental Table S7) generated a diagnostic 808 bp amplicon.

### *In silico* structure and expression analysis

Domain structure of the polypeptide sequence was analyzed at https://www.ebi.ac.uk/interpro/protein/UniProt/. 3D predicted structure was obtained at https://alphafold.ebi.ac.uk/.

Expression of *CUE8/TIC100/EMB1211* in the Arabidopsis GeneAtlas data (Schmid et al., 2005), available at http://jsp.weigelworld.org/AtGenExpress/resources/, was compared with that of a typical photosynthetic protein, *LHCB2* (AT2G05100) and a housekeeping plastid import component, *TOC34* (AT5G05000). Gene expression correlators in relation to development, AtGenExpress tissue compendium data (Schmid et al., 2005) were identified using the BioArray Resource (Toufighi et al., 2005) available at http://bar.utoronto.ca/. Coexpressors were also identified using ATTED-II (Obayashi et al., 2018).

### Chloroplast protein import assays and protein immunoblots

Chloroplasts were isolated from seedlings (Figures 3 and 6) grown *in vitro* to equivalent stages of cotyledons and first leaf pair, for ∼13 days (WT) and 17 days (mutants). Chloroplasts were isolated, examined by phase-contrast microscopy to confirm integrity, and their density quantified. Isolation and import assays using equal numbers of chloroplasts and the RBCS and RPL11 radiolabeled preproteins were carried out as previously described (Kubis et al., 2003). The fraction of preprotein imported, obtained by quantifying on the same import product gel, depended on assay but was at least 10%. Extracts of total chloroplast protein were prepared and equal amounts of protein of WT and mutant were fractionated and subjected to immunoblot using specific antibodies, as described (Kikuchi et al., 2013). Protein samples were denatured at 100°C for 5 min except for the TIC214 (37°C for 30 min) as described (Kikuchi et al., 2013). Antibody dilutions are given in Supplemental Table S5. Quantitation of bands was carried out as described (Kubis et al., 2003) or using ImageJ software.

### RNA extraction and reverse transcription-quantitative PCR analysis

Total RNA was extracted from *in vitro*-grown (under continuous light) 5-day-old WT and 6-day-old *tic100^cue8^* seedlings. Age differences other than 24 h could have resulted in spurious circadian effects. Nucleic acid extraction and quantitation, cDNA synthesis, reverse transcription-quantitative-PCR (RT-qPCR) amplifications, assessment of product quality, and quantitation of expression in the mutant relative to that in the pOCA108 WT were carried out as previously described (Loudya et al., 2020). Primer pairs for RT-qPCR are listed in Supplemental Table S6.

### Mutagenesis and isolation of *soh1*

The *tic100^cue8^* seeds (over 5,000) were mutagenized as described (Lopez-Juez and Hills, 2011) using 50 mM ethyl methanesulfonate for 4 h. About 5,000 healthy M1 *tic100^cue8^* plants (carrying heterozygous mutations) were grown as 50 pools. A putative suppressor in the M2 population from pool 18 was isolated several times and found to have a dramatic phenotype after 2 weeks on soil, which was confirmed by genotyping for the *tic100^cue8^* mutation. The protochlorophyllide and chlorophyll content in its M3 progeny seedlings further showed a clear suppression of *tic100^cue8^*. Genetic analysis of a backcross led to the conclusion of a semi-dominant suppressor mutation. Pair-wise crosses of these suppressors from pool 18 showed them to be allelic.

### Protochlorophyllide and chlorophyll content

Pigments were extracted in dimethyl formamide and quantitation was carried out by spectrophotometry or spectrofluorimetry as previously described (López-Juez et al., 1998; Vinti et al., 2005).

### Mapping by sequencing of the *soh1* mutation

The *soh1* mutation was identified by short-read mapping of a DNA pool from 150 backcrossed BC1F2 (see Supplemental Figure S5) recombinant *tic100^cue8^* phenotypes (F), as well as 100 unmutagenized *tic100^cue8^* WTs (P1) and 100 homozygous *soh1* (P2) parents. Sequencing was carried out at the Oxford Genomics centre, Wellcome Trust Centre for Human Genetics (http://www.well.ox.ac.uk/ogc/) and mapping-by-sequencing was performed using the SHOREmap analysis package (http://bioinfo.mpipz.mpg.de/shoremap/guide.html). To narrow the region, filters were set for quality reads (>100) and indels were included to make sure the polymorphisms of Bensheim were not considered as causal mutations. To identify the semi-dominant mutation, a mapping strategy was designed to first compare the polymorphisms in the *tic100^cue^^8^ soh1* parent (P2, test) caused by mutagenesis and that are absent in the *tic100^cue8^* parent (P1, reference) which gave list A. Second, the polymorphisms (induced mutations) in the backcrossed F2 *tic100^cue8^* population which are absent in P1 resulted in list B. In the last step, list A was used as a test and list B as a reference to find out the EMS-induced true SNPs.

### Gene cloning and generation of transgenic plants

Gene cloning was performed using Gateway Technology (Invitrogen/Thermo Fisher Scientific, Hemel Hempstead, UK). The primers used for the generation of transgenic plants and transient assays are listed in the Supplemental Table S7. The full coding sequences (CDSs) of *tic100^soh1^* and *tic100^cue8^* genes were PCR amplified from the cDNA of the respective Arabidopsis genotypes (Bensheim). The CDSs from entry and destination vectors were confirmed by sequencing (Eurofins Genomics, Constance, Germany) and transformed into the *tic100^cue8^* mutant using *Agrobacterium*-mediated transformation (floral dipping). At least 10 T1 plants resistant on BASTA plates were genotyped in each case (35S:*tic100^soh1^*and 35S:*tic100^cue8^*) and confirmed to carry the transgene.

### Subcellular localization of TIC100 fluorescent protein fusions

To study the protein localization using YFP fluorescence, the CDSs of *TIC100, tic100^cue8^*, and *tic100^soh1^* genes were PCR-amplified without the stop codon from the cDNA of their Arabidopsis parent (Bensheim genotype). The CDSs were introduced into the entry vector, sequenced, and later subcloned into the plant expression vector p2GWY7 carrying a C-terminal YFP tag (Karimi et al., 2005). Protoplast isolation and transfection assays were carried out as described previously (Wu et al., 2009). Plasmid DNA (5 μg) was transfected to 10^5^ protoplasts (0.1 mL of protoplast suspension) isolated from healthy leaves of Arabidopsis Col-0.

The YFP fluorescence images were captured using a Leica TCS SP5 microscope as described previously (Ling and Jarvis, 2015). Images shown represent results of at least two independent experiments showing the same result.

### Lincomycin treatment and associated immunoblotting

Seeds were plated and seedlings grown *in vitro* as indicated above without lincomycin. For lincomycin treatment, seeds were plated on a sterile, fine nylon mesh overlaying MS medium with 1% sucrose for 36 h, at which time the mesh with germinating seeds was transferred to new medium containing in addition 0.5 mM lincomycin, where they continued to grow. Seedlings were harvested for transcripts’ analysis at comparable developmental stages: 5 days for WT, 6 days for *tic100^cue8^* and *tic100^soh1^* and 7 for *tic100^soh1^ gun1*, with two additional days for protein analysis in each case. Total protein extraction used a urea/acetone powders method as described (Loudya et al., 2021). The nitrocellulose membrane with proteins was blocked using Intercept—TBS (LI-COR Biosciences, Cambridge, UK) blocking buffer. The primary antibody against FtsH2 (Var2) and the IRDye 800CW Goat anti-Rabbit IgG secondary antibody were diluted in Intercept buffer (with 0.05% Tween 20). Proteins were detected and imaged with an Odyssey (LI-COR) DLx Imaging System. The results shown are representative of two independent experiments.

### Quantitation of RNA editing

Monitoring and quantitation of editing of two chloroplast mRNAs were carried out as previously described (Loudya et al., 2020).

### Statistical analyses

Averages and standard errors of the mean are indicated. Regressions, chi-squared, Student’s *t* tests (two-tailed), and analysis of variance followed by Dunnett’s tests were carried out in Microsoft Excel, with plug-ins from Real-Statistics.com, for data using the numbers of replicates indicated for each experiment. For morphological parameters, chloroplast quantitative data, chloroplast preparations, import assays, immunoblots, and gene expression assays, the number of samples represent independent biological replicates.

## Accession numbers

Sequence data from this article can be found in the Arabidopsis Genome Initiative or GenBank/EMBL databases under the following accession numbers: *TIC100* (AT5G22640), *GUN1* (AT2G31400), *TIC20-I* (AT1G04940), *TIC20-IV* (AT4G03320), *TIC56* (AT5G01590), *TIC214* (ATCG01130), *TIC110* (AT1G06950), *TIC40* (AT5G16620), and FTSH2 (AT2G30950).

## Supplemental data

The following materials are available in the online version of this article.

**Supplemental Figure S1.** Mutation of *CUE8* delays root development, in a way which can be partly but not fully rescued by growth on sucrose.

**Supplemental Figure S2.** Complementation of *cue8* by genomic DNA containing *TIC100*.

**Supplemental Figure S3.** Developmental expression of *TIC100*, in relation to that of a characteristic photosynthesis-associated and a characteristic plastid housekeeping protein nucleus-encoded gene.

**Supplemental Figure S4.** Chloroplasts of *tic100^cue8^* exhibit reduction specifically in 1-MDa complex component proteins.

**Supplemental Figure S5.** Semidominant phenotype of the *soh1* mutation, and the mapping strategy for gene identification.

**Supplemental Figure S6.** Overexpression of *tic100^cue8^* in the *tic100^cue8^* mutant does not suppress the mutant phenotype.

**Supplemental Figure S7.** Localization of the TIC100 protein, in its WT, TIC100^cue8^, and double-mutated TIC100^soh1^ forms, to the cytoplasm and the chloroplast periphery of transformed, over-expressing intact protoplasts.

**Supplemental Figure S8.** Expression of *TIC*/*TOC* genes in *tic100^cue^^8^ gun1* and *gun1* seedlings, measured relative to their expression in the WT.

**Supplemental Table S1.** List of polymorphic markers used for map-based cloning of *CUE8.*

**Supplemental Table S2**. Analysis of the genomic region containing the *CUE8* gene, and strategies used to rule out alternatives.

**Supplemental Table S3.** Polymorphisms in the *TIC100* sequence between the different genotypes and mutants.

**Supplemental Table S4.** Primers used for genotyping the point mutants by dCAPS/CAPS.

**Supplemental Table S5.** Antibody dilutions used in immunoblotting.

**Supplemental Table S6.** Primers used for RT-qPCR.

**Supplemental Table S7.** Primers used for gene cloning and transgenic approaches.

**Supplemental Data Sets S1 and S2.** Developmental expression co-regulators of *CUE8* identified using Arabidopsis Gene Atlas data, and expression co-regulators according to ATTED-II.

## Supplementary Material

koac153_Supplementary_Data

## Notes

N.L., D.P.F.M., J.B., R.P.J., and E.L.-J. designed the research. D.P.F.M. cloned the *CUE8* gene. N.L. isolated *soh1* and cloned the gene with assistance from S.M.A. N.L. and J.B. performed *in vitro* import assays and associated immunoblot experiments. N.L. performed all microscopy analysis, gene expression quantitation, cloning of fusion proteins, subcellular localization, lincomycin-associated immunoblot, and RNA editing assays. N.L. and E.L.-J. performed genetics and coexpression analyses. N.L., R.P.J., and E.L.-J. wrote the manuscript. P.F.D., R.P.J., and E.L.-J. supervised the project.

The author responsible for distribution of materials integral to the findings presented in this article in accordance with the policy described in the Instructions for Authors (https://academic.oup.com/plcell) is: Enrique López-Juez (e.lopez@rhul.ac.uk).
