# Supplementary material for: Mutations in the chloroplast inner envelope protein TIC100 impair and repair chloroplast protein import and impact retrograde signaling
Source: Plant Cell. 2022 May 30;34(8):3028–46. doi: 10.1093/plcell/koac153 (PMC9338805; doi:10.1093/plcell/koac153)
Supplement: koac153_Supplementary_Data [file koac153_Supplementary_Data.zip › koac153-suppl_data/TIC100 Loudya 2022 Suppl Figs 18 X Suppl Tables 17.pdf]

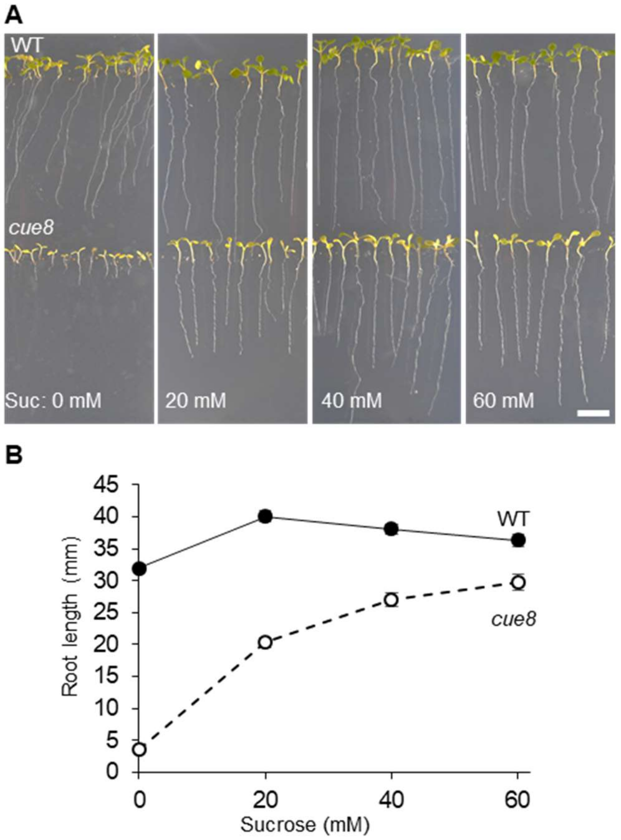

Supplemental Figure S1. Mutation of *CUE8* delays root development, in a way which can be partly but not fully rescued by growth on sucrose. (**A**) 14-day-old seedlings of *cue8* and WT grown on vertical plates, on media containing sucrose at the concentrations indicated. Scale bar: 1 cm. (**B**) Measurement of root length of seedlings grown as above. Error bars represent s.e.m. (n ≥ 30). Values for *cue8* were significantly different to those of WT at each sucrose concentration (Student's t-test, p<0.001). Supports Figure 1.

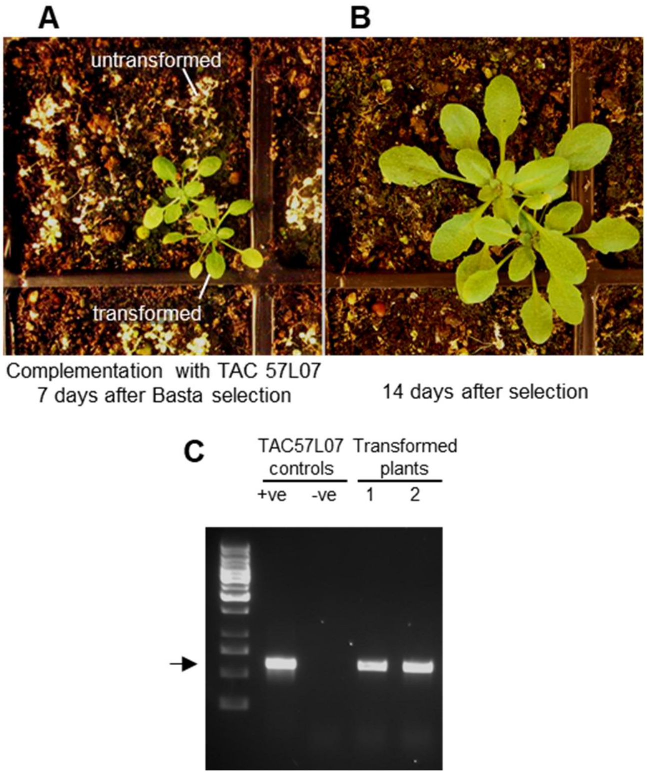

Supplemental Figure S2. Complementation of *cue8* by genomic DNA containing *TIC100*. (**A**) pTAC JatY57L07-transformed *cue8* seedlings, sown on soil, 14 days after germination and 7 days after selection with BASTA, as described in Supplementary Materials and Methods. Note BASTA-sensitive bleached seedlings. (**B**) Same seedlings, 21 days after germination. (**C**) Diagnostic PCR amplification from complemented plants (1, 2) with one primer specific to the pYLTAC17 vector and another specific to the genomic region of TAC57L07, confirming presence of an expected 607 bp product. Positive (+ve) control, plasmid DNA harbouring the construct. Negative (-ve) control, DNA from plant prior to transformation. The second and third bands from the bottom of the left lane correspond to 500 and 750 bp respectively. Supports Figure 2.

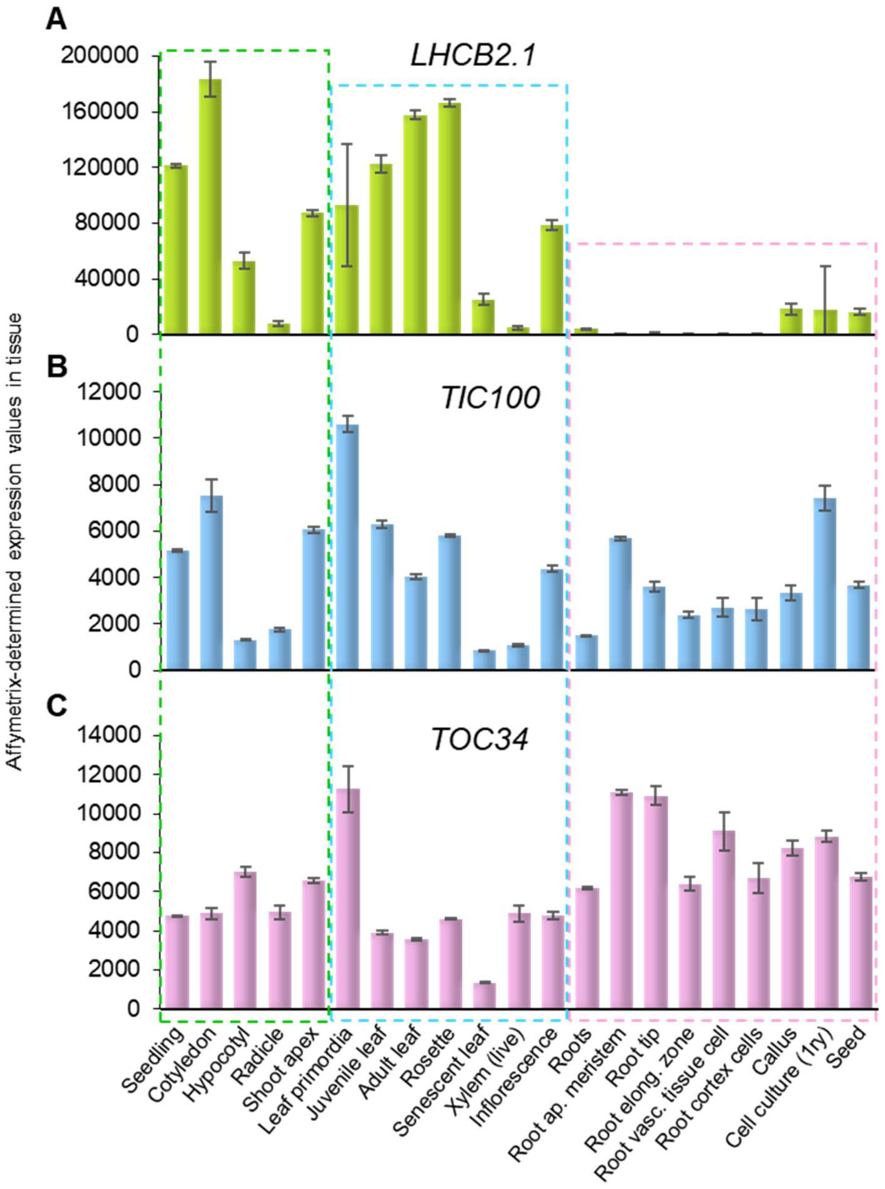

Supplemental Figure S3. Developmental expression of *TIC100*, in relation to that of a characteristic photosynthesis-associated and a characteristic plastid housekeeping protein nucleus-encoded gene. (A) Expression of *LHCB2.1* (AT2G05100), the gene for a photosynthetic antenna polypeptide. **(B)** Expression of *TIC100*. **(C)** Expression of *TOC34* (AT2G05100), a housekeeping plastid import component gene. All developmental expression levels as identified by the Arabidopsis GeneAtlas. Supports Figures 2 and 6.

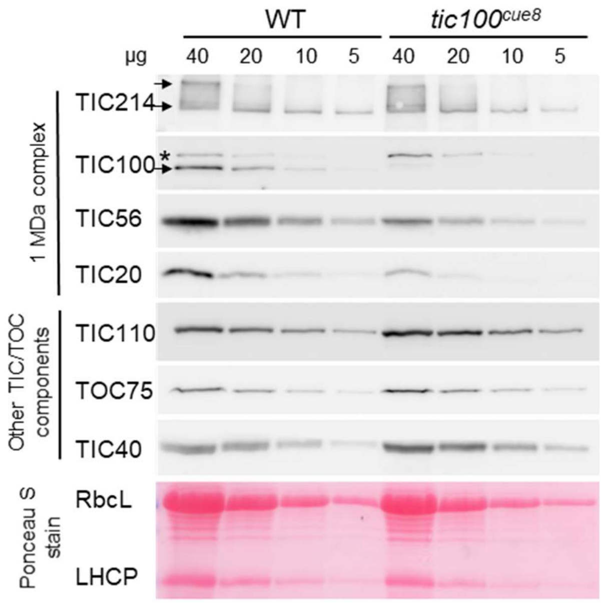

Supplemental Figure S4. Chloroplasts of *tic100^{cue8}* exhibit reduction specifically in 1MDa complex component proteins. Immunoblot analysis of total chloroplast proteins from the *tic100^{cue8}* mutant and wild-type seedlings. The amount of proteins (µg) loaded is indicated above each lane. The antibodies used for the detection of components of the 1 MDa complex (TIC20, TIC56, TIC100 and TIC214) or other chloroplast envelope proteins (TOC75, TIC40 and TIC110), are indicated. In the TIC214 strip both bands, indicated by arrows, correspond to the TIC214 protein, the upper band, indicated by the top arrow, corresponding to an aggregated form of this protein caused by its large size and hydrophobic nature. The asterisk on the TIC100 strip represents a non-specific band, which serves as internal control, while the arrow corresponds to the TIC100 protein. Note the reduced amount of polypeptide components of the 1 MDa complex, in spite of the increased amount of other envelope polypeptides, including the one labelled with an asterisk in the TIC100 strip, in the *cue8* samples. The lower strip represents the Ponceau-stained total protein of one of the replica membranes. Supports Figure 3.

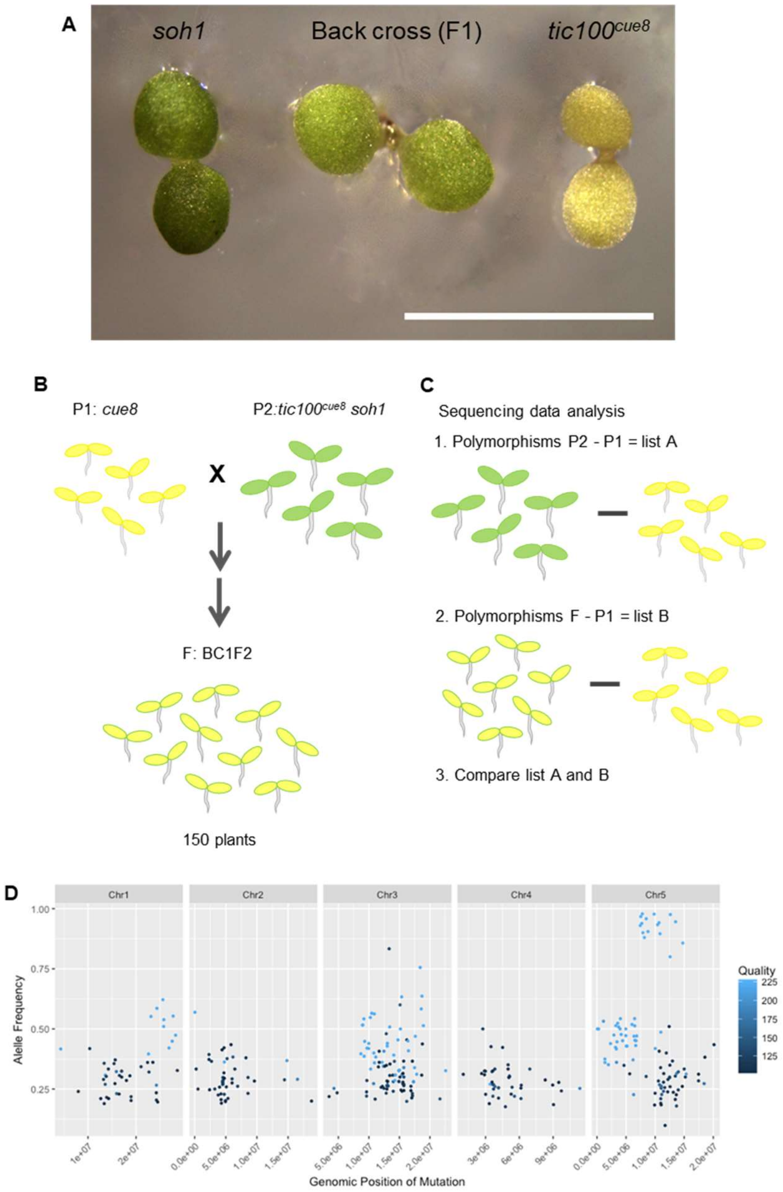

Supplemental Figure S5. Semidominant phenotype of the *soh1* mutation, and the mapping strategy for gene identification. (A) Heterozygous F1 seedlings of a backcross of *soh1* (the *tic100*^{cue8} *soh1* double mutant) to *tic100*^{cue8} are shown, together with a *tic100*^{cue8} and a homozygous *soh1* seedling. Scale bar: 5 mm. Supports Figure 4. **(B)** Strategy followed for the mapping. An F2 population of phenotypically-*cue8* plants was generated from a backcross of the semidominant *soh1* mutant (P2) with its *cue8* parent (P1). This population was high-throughput sequenced in bulk, as were its two parents, before drawing the two lists of polymorphisms to compare. **(C)** Polymorphisms of both parentals (P1 and P2) and the bulked backcrossed F2 segregants population (BC1F2) relative to the Arabidopsis Genome Initiative sequence were compared as shown. **(D)** Visual output of Shoremap software demonstrating the saturation of recombinant mutant allele frequency **(B** shared with **A)** on chromosome 5 around the *TIC100* (AT5G22640) locus. **B** and **C** support Figure 5

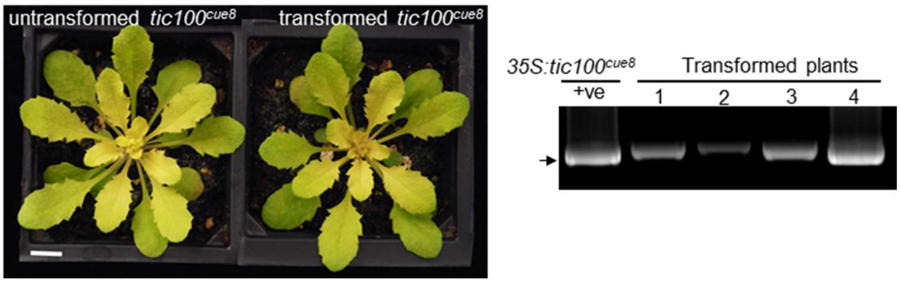

Supplemental Figure S6. Overexpression of *tic100*^{cue8} coding sequence in the *tic100*^{cue8} mutant does not suppress the mutant phenotype. Failure of phenocopying of the suppressor *soh1* mutant by transformation of the single *tic100*^{cue8} mutant with an over-expressed *tic100*^{cue8} sequence driven by the 35S promoter (as seen in 4 independent T1 plants). Plants shown at 40 days of age. Scale bar: 1cm. Gel on the right confirms the genotype of the transformed plants. "+ve": positive genotyping control (bacterial plasmid). Supports Figure 5E.

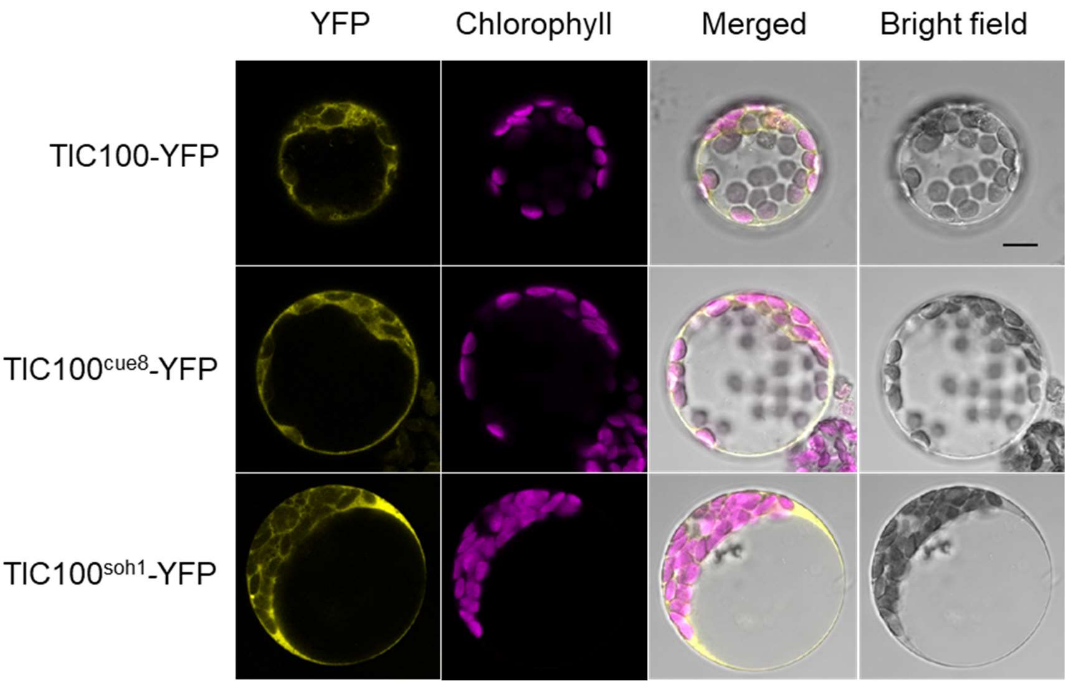

Supplemental Figure S7. Localisation of the TIC100 protein, in its wild type, TIC100^{cue8} and double-mutated TIC100^{soh1} forms, to the cytoplasm and the chloroplast periphery of transformed, over-expressing intact protoplasts. Wild-type protoplasts were transfected with constructs encoding wild-type and mutant forms of TIC100, each one tagged with a C-terminal YFP tag, before observation of the fusion protein using confocal microscopy. Scale bar: 10 μm. Supports Figure 5F.

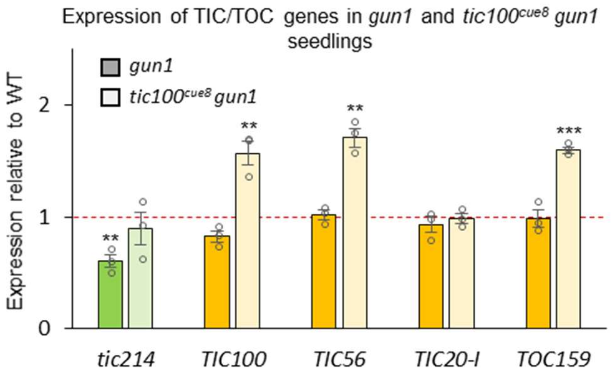

Supplemental Figure S8. The increase in expression of genes for components of the 1MDa complex observed in *tic100^{cue8}* (Fig. 7) is only partially dependent on the action of GUN1, and can be observed to some extent even in GUN1 absence. Expression, measured by quantitative real-time RT-PCR, of *TIC*/*TOC* genes in *tic100^{cue8} gun1* seedlings, measured relative to expression in wild-type seedlings and compared to expression in *gun1*. Note *tic214* is chloroplast-encoded. The presented values are means, and the error bars show s.e.m. of three RNA samples (biological replicates), each with two technical replicates. Asterisks represent significance of difference between mutant and WT (as indicated for Figure 7D, 2-tailed Student's t-test). Dotted lines represent expression in WT. Supports Figure 7.

Supplemental Table S1. List of polymorphic markers used for map-based cloning of *CUE8.* Supports Figure 2

**Primer Pair**	**Chromosome 5 position (bp)**	**Forward Primer (5′ – 3′)**	**Reverse Primer (5′ – 3′)**	**Product (bp)**	**Enzyme associated to SNP**	**Distinguishes ecotype**
541	7021175	GACCCATGTCAGAAGGCAAGC	TTGCGGGATTTGAGAACCTG	531	*Dra*I	Col-0
T10F18-72667-*Cfo*I	7137357	CTGCGATCTCAGTCGGTTAG	GCGAAATTTGGGTTTTACGG	732	*Cfo*I	La-*er*
T10F18-57838-*Hin*fI	7152186	TGAGTGCCACCAATCAGTTC	CTCTGTTTCCTCACTGCAACC	457	*Hin*fI	La-*er*
F13M11-106936-*Hin*fI-*Csp*45I	7168003	TGATGAATTGTGAAGCACTGGTGAG	TCGATTTGAATATGACTGAATGT GAAG	661	*Hin*fI	Col-0
F13M11-106936-*Hin*fI-*Csp*45I	7168003	TGATGAATTGTGAAGCACTGGTGAG	TCGATTTGAATATGACTGAATGT GAAG	661	*Csp*45I	Col-0
T10F18-35093-*Pst*I	7174931	GTTTTGGTCGAGGGTTTGTC	CAGCAGGTCTTCTGGAGTTG	1043	*Pst*I	Col-0
F13M11-96049-*Nde*II	7178890	CAAACCGTAAAATGTCCATAACC	AGCCACGTGTTGCTACTTCC	492	*Nde*II	La-*er*
T1OF18-27618-*Dra*I	7182406	AAAAATCACGGGACGAGTAAAG	ATCGGCAAGAGACGATGTG	820	*Dra*I	La-*er*
T6G21-20198-*Eco*RI	7252484	CATTTCGCTTTTCGCTTTTC	GCTTCGACTACTTCGGCTTG	918	*Eco*RI	La-*er*
T6G12-46724-*Dde*I-*Nde*I	7279010	AATGCTTTAGGGGAGGGTTC	CAGGATACCTCGTGGAGACAG	303	*Dde*I	La-*er*
T6G12-46724-*Dde*I-*Nde*I	7279058	AATGCTTTAGGGGAGGGTTC	CAGGATACCTCGTGGAGACAG	303	*Nde*I	La-*er*
MWD9-6389-*Hin*1I	7367463	CCGTGGAGTTTTCCATCTTC	GCCTCGCATTTTTCTTTGTC	668	*Hin*1I	La-*er*
MWD9-65861-*Dra*I	7426935	TGGTTGTTATGGCCAGCTTC	CAGGCTTTTGCGTGTTTTG	958	*Dra*I	La-*er*
MWD9-73243-*Sac*II-*Cfr*421	7434317	CGTTCCCCATATTTCACTCAC	GTCTTGTCTTGGCTGGCTTC	895	*Sac*II	La-*er*
MWD9-75646-*Ssp*I	7436720	TAAACGGAGATTCAGGAAAATG	TCATTCATACCTTCCCTGTGG	400	*Ssp*I	La-*er*
MQJ16-39094-SSLP	7485589	TAGTGAAACCTTTCTCAGAT	TTATGTTTTCTTCAATCAGTT	100/135	(length polymorph.)	La-*er*
271-*Hin*fI	7530652	TTCGTCATCTGTTTGGGTTG	TCCAACCACTTTCTGTCTTCTG	540	*Hin*fI	Col-0
MDJ22-63740-TaqI	7567266	CATAGTTATGAAGAACTTTGCCTTG	GCCTTCTACGGTTTTTGAGG	602	*Taq*I	Col-0
MRN17-61325-*Dra*I	7666845	GCACGAAAGATATGGGGCTAC	CACTCATGGCTTATTGGATTG	1022	*Dra*I	La-*er*
692	7858086	CACTGCTTTCCGGGATTTAG	AACCGCAGTGGTTTTCTCTG	660	Hsp92II	Col-0

Supplemental Table S2. Analysis of the genomic region containing the *CUE8* gene, and strategies used to rule out alternatives. Supports Figure 2.

Accession	Gene product	Mutation ruled out by complementing TAC?	T-DNA line	Mutation ruled out by T-DNA phenotype?	Mutation ruled out by sequencing?	*CUE8*
AT5G22555	Expressed protein	No			✓	
AT5G22560	Hypothetical protein	No			✓	
AT5G22570	Transcription Factor	No			✓	
AT5G22580	Expressed protein	No			✓	
AT5G22590	Hypothetical protein	No	SALK_144064	✓	✓	
AT5G22600	Expressed protein	No	SALK_093885	No T-DNA	✓	
AT5G22610	F-box family protein	No	SALK_117573	✓		
AT5G22620	Phosphoglycer-ate mutase	No	SALK_012577	✓		
AT5G22630	Prephenate dehydratase	No	SALK_028611	✓		
AT5G22640	TIC100	Complemented by JatY-57L07	SALK_138825	Embryo/ seedling lethal	G2087A (gly366arg)	✓
AT5G22650	Histone deacetylases				✓	
AT5G22660	F-box family protein				✓	
AT5G22670	F-box family protein				✓	
AT5G22680	Hypothetical protein				✓	
AT5G22690	Disease resistance protein		SALK_039393	✓		
AT5G22700	F-box family protein		SALK_009942	✓		
AT5G22720	F-box family protein		SALK_002785	✓		
AT5G22730	F-box family protein				✓	
AT5G22740	Cellulose synthase		SALK_149092	✓		

Supplemental Table S3. Polymorphisms in the *TIC100* sequence between the different genotypes and mutants. Supports Figure 2.

Exon (upper case), intron (low case). In addition to the G366R mutation in *cue8* and the additional R345Q mutation in *cue8 soh1*, ten other polymorphisms were observed against the Arabidopsis Genome Initiative (Col) sequence. Red highlight: mutation in *cue8*. Purple: second mutation in *cue8 soh1*. Yellow, blue and green: polymorphisms between *cue8*/*cue8 soh1*/pOCA108 and Col within introns (yellow) and exons (blue, green). Two polymorphisms in exons confer silent changes (blue), while the third (green) results in a single, conservative amino acid substitution (A820V), distinguishing pOCA108 and Col.

**Sequence differences**	**Found in**	**Position in relation to ATG in Col-0**	**Nucleotide change**	**Significance to protein**
Col different from pOCA108	Intron 2	1181	tggta → tgtta	None
		1207	atcac → attac	
		1214	aataa → aagaa	
		1246	attgt → atcgt	
	Intron 5	1987	tagat → tatat	
	Intron 9	2950	at-tt → atttt	
	Intron 10	3740 - 3741	ctct → c--t	
pOCA108 and *cue8* different from *cue8 soh1*	EXON 5	1867	TCGAA → TCAAA	R345Q *soh1* point mutation
pOCA108 different from *cue8* and *cue8 soh1*	EXON 6	2087	TTGGG → TTAGG	G366R *cue8* point mutation
Col different from pOCA108	EXON 10	3464<br>3551	TTAAC → TTGAC<br>CAAGA → CAGGA	None
Col different from pOCA108	EXON 11	4062	CGCAT → CGTAT	A820V polymorphism

Supplemental Table S4. Primers used for genotyping the point mutants by dCAPS /CAPS.

Mutant	Forward/ reverse	Sequence	Enzyme digestion	Band size (bp)
cue8	cue8-dCAPS_F	ACATCTTAATGGTAACGCAGGGTAGATTCT ACCT	Dde1 (digests *cue8*)	*cue8* (164) *CUE8* (197)
	cue8-dCAPS_R	TCCGCCAAACCAGAATGCAGCTG		
soh1	soh1-CAPS_F	GAGAAACCGTGAGTCTGCGA	BsmI (digests *SOH1*)	*soh1* (637 + 176) *SOH1* (358 + 280 + 176)
	soh1-CAPS_R	AGAGGGTCCTGCACTGATCT		
gun1-1	gun1-dCAPS_F	TAACTATTGCTAAGAGGATTTTCGAAACAG	AluI (digests *GUN1*)	*gun1* (99) *GUN1* (69 + 30)
	gun1-dCAPS_R	CACTTCTCCCATAAGCGCTGA		

Supplemental Table S5. Antibody dilutions used in immunoblotting.

Antibody	Dilution	Source
Anti-TIC214	1:5,000	Kikuchi et al., 2013
Anti-TIC100	1:5,000	
Anti-TIC56	1:5,000	
Anti-TIC20	1:50	
Anti-TIC110	1:5,000	Ling et al., 2012
Anti-TIC40	1:1,000,000	
Anti-TOC75	1:1,000	
Anti-HSP70	1:5,000	
Anti-RPL2	1:5,000	Subramanian lab, Berlin
Anti-FtsH2 (Anti-Var2)	1:5,000	Sakamoto lab, Okayama

Supplemental Table S6. List of primers used for quantitative real-time RT-PCR.

Gene	Locus	Forward / Reverse	Primer sequence
UBQ10	AT4G05320	UBQ10_F	GGAGGATGGTCGTACTTTGG
		UBQ10_R	TCCACTTCAAGGGTGATGGT
TOC159	AT4G02510	TOC159_F	AGAGGCGATTTAGCCCTTGGAG
		TOC159_R	CCTGCACGAAGCGCAATCTTTG
TIC100	AT5G22640	TIC100_F	GAGATGATACAGCAAGAACT
		TIC100_R	AATTCTTCATCCATATCTTC
TIC56	AT5G01590	TIC56_F	AGGAGTGTCATGAGGCTATTCCG
		TIC56_R	AGCTTCTGGCCTACTCGAACAC
TIC20-I	AT1G04940	TIC20-I_F	CGTTTGTCTGTGATGCTGCC
		TIC20-I_R	GAGGAGTCATAACGATCCAATGT
TIC20-IV	AT4G03320	TIC20-IV_F	TTGAGAAGACACCGGAGACC
		TIC20-IV_R	AACGTCCTCCACCACCATTC
tic214	ATCG01130	TIC214_F	AGAATCGGCCGGTCAAGTAGAAC
		TIC214_R	AATCGAGCTGCTTCGGGATTTC
LHCB1.2	AT1G29910	LHCB1_F	CCGATCCAGTCAACAACAAC
		LHCB1_R	TCAAACCATCACATACAACCTTC
RBCS	AT1G67090	RBCS_F	ACTTCCATCACAAGCAACGG
		RBCS_R	CGGAATCGGTAAGGTCAGGA
CA1	AT3G01500	CA1_F	GAAGGACTTGTGAAGGGAACA
		CA1_R	TTTAACAGAGCTAGTTTCGGAGAG

Supplemental Table S7. Primers used for gene cloning and transgenic approaches.

Primer	Sequence	Used to
CUE8_CDS_Topo_F	CACCATGGCTAACGAAGAACTCAC	PCR amplify the CUE8/TIC100 coding sequence using TOPO cloning for complementation.
CUE8_CDS_Topo_R	AGAGACTCAAGACACAGCAGGA	
CUE8_CDSgate_F	TGCCCAGCTATCTGTCACTTC	Genotype the plants transformed using pB7WG2 vector.
CUE8_CDSgate_R	CTTCCAACGTTCTGGGTCTC	
pYLTAC17_F	AATCCTGTTGCCCDCCTTG	Genotyping the bacteria and the transformed plants with the TAC JatY-57L07.
57L07-FOR_R	GTCTGAGCCAGAGCCAGAGCTTGAGG	
TIC100_CDS_F	ATGGCTAACGAAGAACTCAC	PCR amplify the *cue8* or *cue8 soh1* coding sequences
TIC100_CDS_R	TCAAGACACAGCAGGAGTCT	
attB1-TIC100_F	GGGGACAAGTTTGTACAAAAAAGCAGGC TTCATGGCTAACGAAGAACTCAC	PCR amplify and add the attB1B2 sites to *cue8* or *cue8 soh1* coding sequences
attB2-TIC100_R	GGGGACCACTTTGTACAAGAAAGCTGGG TCTCAAGACACAGCAGGAGTCT	
TIC100_seq_ F2	AGGTTCCAAGCTTGAAGCT	Check the complete CDSs after sequencing
TIC100_seq_R2	TTGAAGGATCCACTTCTTCT	
TIC100 3'F	GTATCATCATCTTCTTCTCC	Check the *TIC100 / cue8 / soh1* ends and orientation in the vector during sequencing.
TIC100 5'R	GATGTAGAAATCGTCGCCG	
TIC100_cDNA_no stop_R	AGACACAGCAGGAGTCTCAG	PCR amplify the *TIC100*/ *cue8* /*soh1* coding sequences without stop codon for the YFP constructs (combination with TIC100_cDNAF)
attB2-TIC100_no stop_R	GGGGACCACTTTGTACAAGAAAGCTGGG TCAGACACAGCAGGAGTCTCAG	PCR amplify and add the attB1B2 sites to *cue8*/*soh1* coding sequences (combination with attB1-TIC100_F)
pB2GW7_35S_F	ACGCACAATCCCACTATCCT	Sequence analysis and to genotype the plants transformed using pB2GW7 vector.
pB2GW7_35S_R	CAACACATGAGCGAAACCCT	
p2GWY7_35S_F	ACGCACAATCCCACTATCCT	Sequence analysis and to genotype of the YFP destination vector.
p2GWY7_35S_R	CAACACATGAGCGAAACCCT	
